# Simultaneous Binding of Multiple EF-Tu Copies to Translating Ribosomes in Live *Escherichia coli*

**DOI:** 10.1128/mBio.02143-17

**Published:** 2018-01-16

**Authors:** Mainak Mustafi, James C. Weisshaar

**Affiliations:** a Department of Chemistry, University of Wisconsin—Madison, Madison, Wisconsin, USA; Massachusetts Institute of Technology

**Keywords:** EF-Tu, binding to ribosome, live *E. coli*, single-molecule tracking

## Abstract

In bacteria, elongation factor Tu is a translational cofactor that forms ternary complexes with aminoacyl-tRNA (aa-tRNA) and GTP. Binding of a ternary complex to one of four flexible L7/L12 units on the ribosome tethers a charged tRNA in close proximity to the ribosomal A site. Two sequential tests for a match between the aa-tRNA anticodon and the current mRNA codon then follow. Because one elongation cycle can occur in as little as 50 ms and the vast majority of aa-tRNA copies are not cognate with the current mRNA codon, this testing must occur rapidly. We present a single-molecule localization and tracking study of fluorescently labeled EF-Tu in live *Escherichia coli*. Imaging at 2 ms/frame distinguishes 60% slowly diffusing EF-Tu copies (assigned as transiently bound to translating ribosome) from 40% rapidly diffusing copies (assigned as a mixture of free ternary complexes and free EF-Tu). Combining these percentages with copy number estimates, we infer that the four L7/L12 sites are essentially saturated with ternary complexes in vivo. The results corroborate an earlier inference that all four sites can simultaneously tether ternary complexes near the A site, creating a high local concentration that may greatly enhance the rate of testing of aa-tRNAs. Our data and a combinatorial argument both suggest that the initial recognition test for a codon-anticodon match occurs in less than 1 to 2 ms per aa-tRNA copy. The results refute a recent study (A. Plochowietz, I. Farrell, Z. Smilansky, B. S. Cooperman, and A. N. Kapanidis, Nucleic Acids Res 45:926–937, 2016, https://doi.org/10.1093/nar/gkw787) of tRNA diffusion in *E. coli* that inferred that aa-tRNAs arrive at the ribosomal A site as bare monomers, not as ternary complexes.

## IMPORTANCE

Ribosomes catalyze translation of the mRNA codon sequence into the corresponding sequence of amino acids within the nascent polypeptide chain. Polypeptide elongation can be as fast as 50 ms per added amino acid. Each amino acid arrives at the ribosome as a ternary complex comprising an aminoacyl-tRNA (aa-tRNA), an elongation factor called EF-Tu, and GTP. There are 43 different aa-tRNAs in use, only one of which typically matches the current mRNA codon. Thus, ternary complexes must be tested very rapidly. Here we use fluorescence-based single-molecule methods that locate and track single EF-Tu copies in *E. coli*. Fast and slow diffusive behavior determines the fraction of EF-Tu copies that are ribosome bound. We infer simultaneous tethering of ~4 ternary complexes to the ribosome, which may facilitate rapid initial testing for codon matching on a time scale of less than 1 to 2 ms per aa-tRNA.

## INTRODUCTION

In protein synthesis, the elongation cycle comprises an elaborate sequence of steps (1, 2). After an aminoacyl-tRNA (aa-tRNA) binds to the ribosome, it is tested for a match between its anticodon and the current mRNA codon. When a cognate aa-tRNA is found, peptide bond formation occurs and the tRNAs and mRNA translocate through the ribosome, enabling the cycle to begin again. In bacteria, the codon recognition step is catalyzed by elongation factor Tu (EF-Tu), a GTPase. Its eukaryotic homologue is called eEF-1A (3). The translocation step is catalyzed by a second GTPase called elongation factor G (EF-G) (2).

**FIG 1:**
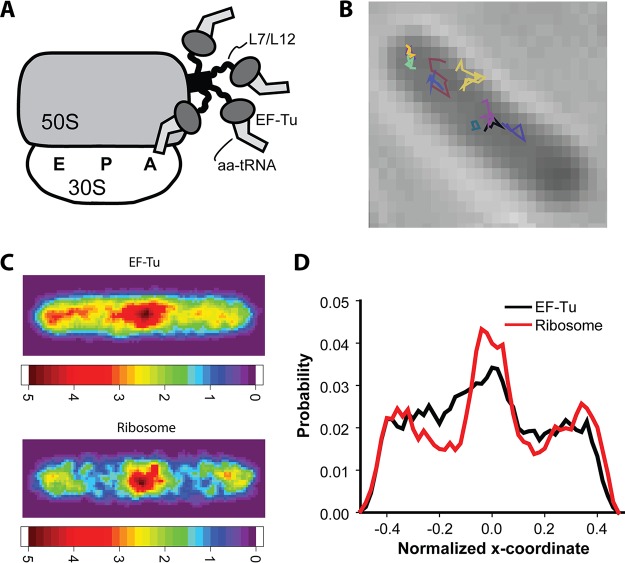
(A) Schematic diagram showing four ternary complexes bound to the four L7/L12 units on the stalk of a 70S ribosome. One of the ternary complexes is also bound to the A site for codon testing (Based on the model of reference 4). We emphasize that while biochemical studies support binding of the ternary complex to L7/L12, the stalk is highly mobile in all structural studies to date (2). (B) Several single-molecule trajectories of EF-Tu–mEos2 plotted in different colors and superimposed on the phase-contrast image of the same cell. (C, top) Composite spatial distribution heat map of EF-Tu–mEos2 for 4,221 localizations from 201 *E. coli* cells of length 4 to 5 µm. Pixels are ~45 by 45 nm. The intensity scale shows relative counts per pixel. (Bottom) Composite spatial distribution heat map of ribosomes (30S–mEos2 labeling) for 1,967 localizations from 108 *E. coli* cells of length 4 to 5 µm. (D) The projected axial distribution of EF-Tu–mEos2 and ribosomes (30S–mEos2) for the same sets of cells used in panels C and D. The distributions are normalized to the same area and plotted on a relative scale of −0.5 to +0.5 for the long axis.

In the standard mechanistic model of *Escherichia coli* translation (1, 2), aa-tRNA binds to the ribosome as a ternary complex: aa-tRNA–EF-Tu(GTP). The ternary complex is recruited to the ribosome by binding to one of four L7/L12 sites that protrude from the stalk of the ribosome, as shown schematically in Fig. 1A (4). L7 is identical to L12, except for an acylated N terminus. Biochemical evidence indicates that the binding interface juxtaposes the C-terminal domain of L7/L12 and domain 1 of EF-Tu (5, 6). The ribosomal stalk thus tethers aa-tRNA copies in close proximity to the ribosomal A site, where they can be tested for a codon match. Under good growth conditions, *E. coli* can carry out elongation at a rate of ~17 to 20 amino acids/s, implying that the mean time to carry out a complete elongation cycle can be as short as 50 ms (7, 8). Since the vast majority of aa-tRNA copies carry a noncognate or near-cognate anticodon that does not match the current mRNA codon (9), testing of individual aa-tRNAs for a codon match must be very rapid. A recent global theory of bacterial metabolism suggested that the diffusive search of EF-Tu for its ribosomal binding site is the step limiting the overall growth rate (10).

The sequence of events leading from the initial binding step to codon recognition and peptide bond formation has been dissected in remarkable detail by a groundbreaking series of rapid-mixing kinetics experiments carried out *in vitro* and summarized in references 1 and 9. Single-molecule studies *in vitro* have helped to further refine the detailed sequence of mechanistic steps (11, 12). The inferred mechanism includes two consecutive stages of codon discrimination: initial selection and subsequent proofreading, with multiple intermediate states delineated for both stages (1). The overall mechanism enables cognate aa-tRNAs to proceed rapidly to accommodation in the A site, while rapidly rejecting noncognate and near-cognate aa-tRNAs. Most recently, a detailed set of *in vitro* transition rates has been optimally scaled to form a theoretical set of *in vivo*, codon-specific transition rates that yield the correct overall translation rate in exponentially growing *E. coli* (9). These optimized *in vivo* transition rates were then used to predict codon-dependent translation speeds, codon-specific translation dynamics, and missense error frequencies. The good agreement of the model predictions with the experiments serves to validate the new method for transforming detailed *in vitro* rates into useful *in vivo* rates.

The *E. coli* ribosomal stalk (schematic in Fig. 1A) comprises the L11 protein, which binds to rRNA and forms the base of the stalk, the protruding L10 protein, which binds to L11 via a flexible connection, and four L7/L12 copies, which bind to L10 as a pair of dimers (4). Each L7/L12 has three domains. The N terminus binds to L10, and a flexible hinge connects the N terminus to the C terminus. A compelling body of biochemical evidence detailed in reference 4 and summarized below indicates that the C-terminal domain of L7/L12 binds to helix D of EF-Tu within the ternary complex. The only structural evidence for L7/L12 binding to EF-Tu comes from a cryo-electron microscopy (cryo-EM) reconstruction at a 1.8-nm resolution (13). The structure suggests a bridge between domain 1 of EF-Tu (the G domain) and the L7/L12 stalk, in agreement with inferences from the biochemical data. A comprehensive model of ribosomal stalk structure and function suggested that the four highly mobile L7/L12 C-terminal domains serve to efficiently recruit ternary complexes to the ribosome and help stabilize the active GTPase conformation of EF-Tu (4). However, there is no crystal structure that reveals the molecular-level details of the initial binding step of the ternary complex to the ribosome. In all high-resolution structural studies to date, the L7/L12 stalk is highly mobile and does not yield discernible electron density (2).

We and others have used live-cell, single-molecule fluorescence methods to study the spatial distribution and diffusive properties of a variety of proteins in *E. coli* (14, 15). In a typical experiment, the protein of interest is expressed from the chromosome as a fusion to a photoconvertible fluorescent protein. A weak laser at 405 nm switches the absorption and emission wavelengths of literally one or two protein copies per cell. A more powerful probe laser then enables selective excitation, localization, and tracking of the sparse photoswitched copies until they photobleach. For high-copy-number proteins, this enables the acquisition of thousands of single-molecule trajectories from each cell over tens of seconds. The spatial localization accuracy is typically a σ value of ~40 to 80 nm, and the temporal resolution can be in the low-millisecond range (16). In favorable cases, the diffusive properties of a single copy can be related to its biochemical function at a given moment in time.

Here we present a single-molecule localization and tracking study of EF-Tu in *E. coli*. EF-Tu is labeled at the C terminus with the 26-kDa (17) photoconvertible fluorescent protein mEos2 (18). Measurement of a large number of short-lived diffusive tracks at 2 ms/frame enables an approximate decomposition of the EF-Tu population into two substates. We call these states “slow” (assigned as copies transiently bound to translating 70S ribosomes, including polysomes) and “fast” (copies not bound to ribosomes, presumably mostly EF-Tu within free ternary complexes). Accordingly, the slow copies (~60%) concentrate in the three ribosome-rich regions where most translation occurs, outside the nucleoids (15, 19).

Combining the new diffusion data with copy number estimates for ribosomes and EF-Tu indicates that the four L7/L12 sites are essentially saturated with EF-Tu copies *in vivo*. This new result corroborates the earlier inference from *in vitro* kinetics measurements that all four *E. coli* L7/L12 sites are actively engaged in recruiting ternary complexes to the ribosome (4). The time scale of binding events indicates that free ternary complexes find translating ribosomes extremely efficiently, in good quantitative agreement with the recent model of *in vivo* kinetics (9). Evidently aa-tRNA copies are tested for a match to the current codon on a time scale of 1 to 2 ms or less, in further agreement with the *in vivo* model. Simultaneous binding of four ternary complexes to each translating ribosome may greatly enhance the rate of testing (4). Finally, the results refute the main conclusion from a recent single-molecule tracking study of tRNA diffusion in *E. coli* (20). That work inferred that most aa-tRNAs are monomeric and freely diffusing, arriving at the ribosomal A site as bare aa-tRNAs, not as ternary complexes.

## RESULTS

### Comparison of axial spatial distributions of EF-Tu and ribosomes.

Essentially identical copies of EF-Tu are expressed by two genes in *E. coli*: *tufA* and *tufB* (21). We have fused the gene coding for the photoconvertible fluorescent protein mEos2 to the C terminus of both endogenous genes within the chromosome in the *E. coli* strain NCM3722 and then moved the fusions to the VH1000 background strain for further study (see Table S1 in the supplemental material).

10.1128/mBio.02143-17.10 TABLE S1  (Part A) Bacterial strains used. The background strain is *E. coli* VH1000. Also shown are the oligonucleotides used (part B) and the sources of copy number estimates relative to ribosomes (part C). Download TABLE S1, PDF file, 0.2 MB. Copyright © 2018 Mustafi and Weisshaar. 2018 Mustafi and Weisshaar This content is distributed under the terms of the Creative Commons Attribution 4.0 International license.

Labeling of all copies of EF-Tu with mEos2 ensures that there is no competition with unlabeled copies. Domain 3 of EF-Tu binds to tRNA and includes the C terminus, but mEos2 is appended on the face opposite to the tRNA binding site. In “EZ rich, defined medium” (EZRDM), the doubling time at 30°C of the modified strain expressing EF-Tu–mEos2 from the chromosome is 60 ± 3 min, compared with 45 ± 2 min (19) for the unlabeled VH1000 background strain (see Fig. S8 in the supplemental material). Evidently the labeling does not greatly affect the functionality of EF-Tu, an essential protein.

Our goal is to use diffusive properties to distinguish ribosome-bound EF-Tu from EF-Tu not bound to ribosomes. The mass of bare EF-Tu–mEos2 is 69 kDa, 26 kDa of which is due to mEos2. The mass of a typical labeled ternary complex, including mEos2 [aa-tRNA–EF-Tu(GTP)-mEos2] is ~95 kDa. We would expect the diffusion coefficients of free ternary complexes (not bound to ribosomes) and of free, bare EF-Tu in the cytoplasm to be similarly fast—perhaps 4 to 8 μm^2^/s (22, 23). Short diffusive trajectories with significant localization error will not be able to distinguish free ternary complexes from bare EF-Tu; we use “fast EF-Tu” to denote a composite of these two species. Below we will argue that a large majority of these fast EF-Tu copies are bound within ternary complexes. In contrast, the ribosome mass is ~2.5 MDa (24, 25) and translating 70S ribosomes in exponentially growing *E. coli* exist primarily as polysomes (15, 19, 26). The mean 70S ribosome diffusion coefficient under these fast imaging conditions is ~0.1 μm^2^/s (supplemental material). EF-Tu copies that are bound to translating 70S ribosomes should diffuse similarly slowly.

It was previously shown that under our moderately fast exponential growth conditions, *E. coli* exhibits strong segregation of the two major nucleoid lobes from the 70S ribosomes (19). The projected axial distribution of ribosomes within the cytoplasm typically has three peaks, with the two nucleoid lobes interleaving three “ribosome-rich regions.” In contrast, free 30S and 50S subunits readily penetrate the nucleoid regions (15, 19, 27). Segregation of 70S ribosomes from the chromosomal DNA may serve to enhance the efficiency of recycling of 30S and 50S subunits and also the efficiency of the search for transcription initiation sites by RNA polymerase. The slowly diffusing EF-Tu ternary complexes bound to 70S ribosomes should also exhibit a three-peaked axial distribution, while rapidly diffusing, free EF-Tu should be distributed more uniformly.

We imaged EF-Tu–mEos2 molecules in cells by photoactivating and locating fluorophores, connecting locations over multiple frames to form trajectories of individual molecules (28). Details are provided in Materials and Methods and the supplemental material. To enable efficient superresolution imaging of rapidly diffusing molecules, the exposure time was 2 ms/frame with continuous laser illumination. The number of switched-on copies per cell was limited to 0 to 2 molecules per frame to avoid spatial overlap of the single-molecule features.

Movie S1 shows typical raw data, and several example trajectories from a single cell are shown in Fig. 1B. In constructing axial spatial distributions that combine data from many cells, we included only cells that were 4 to 5 µm in tip-to-tip length to minimize blurring of features. From 201 such cells, we obtained 4,221 EF-Tu–mEos2 trajectories that lasted at least 6 steps (7 camera frames, or a total duration of 12 ms). All localizations were included in the spatial distributions. The axial and radial cell dimensions were normalized, and the relative molecular positions were pixelated and plotted to obtain a two-dimensional heat map of the EF-Tu spatial distribution (Fig. 1C, top). The map shows that EF-Tu is distributed over the entire cytoplasm, but the distribution is not homogeneous. For comparison, in Fig. 1C (bottom), we show the heat map for ribosomes with the 30S subunit labeled by the endogenously expressed S2-mEos2 protein at the C terminus as before (19) and imaged under the same conditions used for EF-Tu. Again, trajectories of 6 steps or longer in cells 4 to 5 μm in length were included. As shown qualitatively by the heat maps of Fig. 1C and quantitatively in the projected axial distributions of Fig. 1D, ribosomes exhibit substantially greater segregation from the nucleoids than EF-Tu. The total EF-Tu distribution does exhibit three peaks, but they are less sharply defined. This indicates that at a given moment, only a fraction of EF-Tu–mEos2 copies are associated with 70S ribosomes.

10.1128/mBio.02143-17.11 MOVIE S1  Tracking individual EF-Tu molecules tagged with photoconvertible fluorescent protein mEos2 in a single live *E. coli* cell. The movie represents 2 ms/frame with continuous laser exposure; 300 frames of the entire movie are shown. The cell outline (cyan) was obtained from a phase-contrast image. The movie plays back at 3 frames/s. Download MOVIE S1, AVI file, 1.5 MB. Copyright © 2018 Mustafi and Weisshaar. 2018 Mustafi and Weisshaar This content is distributed under the terms of the Creative Commons Attribution 4.0 International license.

### Diffusion of EF-Tu.

**FIG 2:**
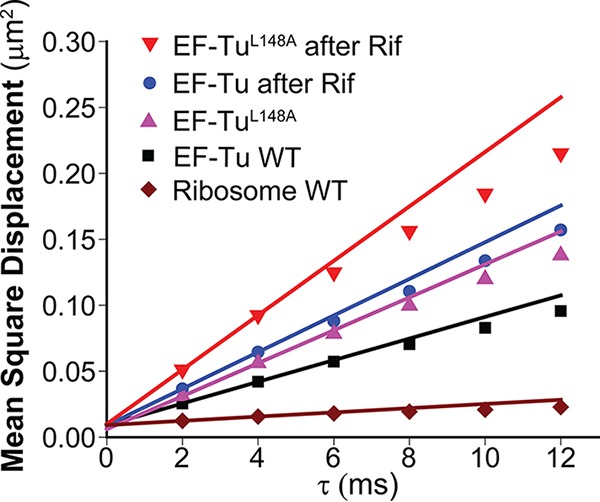
Mean square displacement (MSD) plots for WT EF-Tu, the mutant form EF-Tu^L148A^, and ribosomes under normal growth conditions and with Rif treatment as indicated. Slopes from the first two points yield population-averaged diffusion coefficient estimates as follows: WT EF-Tu, 2.02 ± 0.19 µm^2^/s; WT EF-Tu after Rif treatment, 3.5 ± 0.4 µm^2^/s; mutant EF-Tu^L148A^, 3.1 ± 0.3 µm^2^/s; mutant EF-Tu^L148A^ after Rif treatment, 5.2 ± 0.4 µm^2^/s; and ribosomes (30S–mEos2 labeling), 0.4 ± 0.1 µm^2^/s.

For the diffusion study, we used 1,912 trajectories of duration 6 steps or longer obtained from 118 different cells. Longer trajectories were truncated at 6 steps. The exposure time was 2 ms/frame. The mean diffusion coefficient, *D*_mean_, can be estimated from a plot of the two-dimensional mean-square displacement versus lag time, MSD(τ), using the slope of the first two data points. This provides a population-weighted average of diffusion coefficients over the different states of the molecule. The MSD slope accounts for localization error, but does not account for confinement effects. In Fig. 2, we compare MSD plots for wild-type (WT) EF-Tu and ribosomes. The mean diffusion coefficients are 2.02 ± 0.19 µm^2^/s for EF-Tu and 0.4 ± 0.1 μm^2^/s for ribosomes. The mean value for EF-Tu is consistent with the existence of at least two diffusive states: a fast, rapidly diffusing EF-Tu state and a slow, ribosome-bound state. The intercept of the MSD plot provides an estimate of the mean localization accuracy σ value of ~60 nm (29).

**FIG 3:**
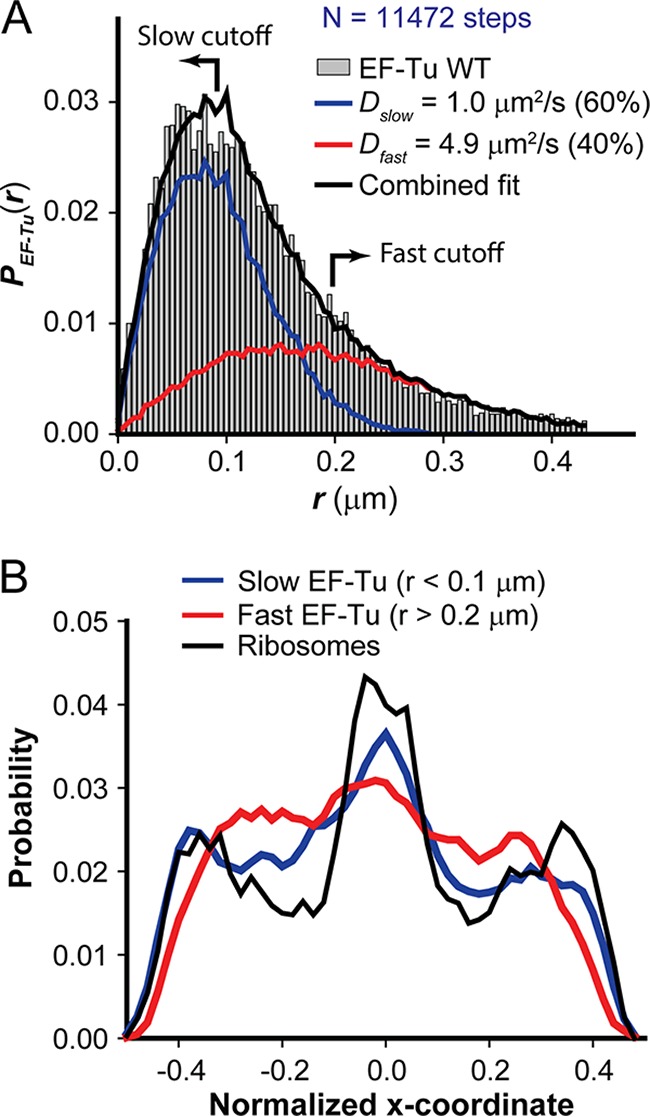
(A) The experimental distribution of single-step displacements *P*_EF-Tu_(*r*) (gray histogram) for 11,472 2-ms steps for WT EF-Tu. The solid black line shows the best-fit model using two static states: “slow” (blue) and “fast” (red). Model parameters: *f*_slow_ = 0.6, *D*_slow_ = 1.0 µm^2^/s, *f*_fast_ = 0.4, and *D*_fast_ = 4.9 µm^2^/s. (B) Axial distributions of predominantly slow (blue) and fast (red) single-step displacements of WT EF-Tu in comparison with ribosome axial distribution (30S-mEos2 labeling [black]). The cutoffs chosen to separate slow (<0.1-µm) and fast (>0.2-µm) single-step displacements are indicated by the arrows in panel A. The distributions are normalized to the same area and plotted on a relative scale of −0.5 to +0.5 for the long axis.

In order to quantify the fraction of ribosome-bound EF-Tu copies, the same truncated trajectories were divided into individual steps with Δ*t* = 2 ms between camera frames. This attempts to isolate short time intervals during which EF-Tu remains in one particular diffusive state (16). The resulting distribution of experimental single-step displacements, *P*_EF-Tu_(*r*), is shown for 11,472 individual steps in Fig. 3A. We analyze such *P*(*r*) distributions by comparison with a large number of simulated random walk trajectories that incorporate dynamic localization error σ and confinement within a spherocylinder that mimics the dimensions of an *E. coli* cell. Details are provided in the supplemental material. For each chosen model diffusion coefficient, *D*, and measurement error, σ, the simulations provide a numerical function we call *P*_model_(*r*; *D*). We attempt to fit the experimental distribution *P*(*r*) using least squares to a single population or to a weighted average of two static populations. The goodness of each fit was judged by the reduced chi-square statistic, χ_ν_^2^, which should be approximately 1 for an appropriate model function (30). For a one-state model, the only fitting parameter is *D*. For unconstrained models, including two static (nonexchanging) states, the fitting function is the linear combination *P*_model_(*r*) = *f*_slow_*P*(*r*; *D*_slow_) + (1 − *f*_slow_)*P*(*r*; *D*_fast_). Here the three fitting parameters are *D*_fast_, *D*_slow_, and the fractional population *f*_slow_, which in turn fixes *f*_fast_ = (1 − *f*_slow_).

One-component fits to the *P*_EF-Tu_(*r*) were poor, with minimum χ_ν_^2^ = 9.7 (see Fig. S1B in the supplemental material). Fits to two nonexchanging diffusive states were substantially better. The best value of χ_ν_^2^ was 1.24, obtained using model parameters *f*_slow_ = 0.60 ± 0.05, *D*_slow_ = 1.0 ± 0.2 µm^2^/s, *f*_fast_ = 0.40 ± 0.05, and *D*_fast_ = 4.9 ± 1.2 µm^2^/s (Table 1). The best-fit two-state model result is plotted in Fig. 3A and resolved into the two separate contributions. The parameter uncertainties are based on the range of parameters that return reduced chi-square values within 0.5 units of the best value, as detailed in the supplemental material. Parameter sets with χ_ν_^2^ values still larger were judged by eye to be qualitatively poor. The best two-component constrained fit to *P*_EF-Tu_(*r*) with *D*_slow_ fixed at 0.1 μm^2^/s (to match the slow, 70S component of the ribosome diffusion data) has χ_ν_^2^ = 2.5 (Fig. S1A), which is much worse than the global best-fit value of 1.24. Our constrained search for three-component fits did not reduce χ_ν_^2^ significantly (supplemental material, Fig. S1C).

10.1128/mBio.02143-17.2 FIG S1  Different fitting models for WT EF-Tu single-step displacement probability distribution *P*_EF-Tu_(*r*). (A) Two static states with *D*_slow_ fixed at 0.1 µm^2^/s (corresponding to the estimated 70S ribosome diffusion coefficient). Best-fit *D*_fast_ and fractions as shown yield χ_ν_^2^ = 2.5, indicating the model is inadequate. (B) Single static state. The minimum χ_ν_^2^ is 9.7, corresponding to *D*_fit_ = 1.0 µm^2^/s. (C) three static states with *D*_slow_ and *D*_fast_ fixed at 0.1 and 4.9 µm^2^/s, respectively. The best-fit *D*_medium_ and fractions are shown. The minimum χ_ν_^2^ is 1.25, which is not an improvement over the best two-state model of Fig. 3. Download FIG S1, TIF file, 18 MB. Copyright © 2018 Mustafi and Weisshaar. 2018 Mustafi and Weisshaar This content is distributed under the terms of the Creative Commons Attribution 4.0 International license.

**TABLE 1:** Summary of best-fit diffusion coefficients and fractional populations

EF-Tu or ribosome type^a^	*D*_mean_ (µm^2^/s)^b^	*f*_slow_^c^	*D*_slow_ (µm^2^/s)	*D*_fast_ (µm^2^/s)
Normal growth conditions				
EF-Tu WT^d^	2.02 ± 0.19	0.60 ± 0.05	1.0 ± 0.2	4.9 ± 1.2
Ribosome WT^e^	0.4 ± 0.1	0.7 ± 0.05	0.1 ± 0.1	1.2 ± 0.5
EF-Tu^L148A^ mutant^d^	3.1 ± 0.3	0.3 ± 0.05	1.2 ± 0.5	4.5 ± 1.0
After Rif treatment				
EF-Tu WT^d^	3.5 ± 0.4	0.35 ± 0.05	1.5 ± 0.5	4.9 ± 1.5
EF-Tu^L148A^ mutant^d^	5.2 ± 0.4	0.1 ± 0.05	1.9 ± 1.2	5.6 ± 1.2

a Normal growth conditions were used, except for measurements after rifampin (Rif) treatment as noted.

b Mean diffusion coefficient estimated from first two points of MSD plot (Fig. 2).

c Best-fit fractional population of the more slowly diffusing state. The fractional population of the more rapidly diffusing state is *f*_fast_ = 1 – *f*_slow_.

d C terminus labeled with mEos2.

e 30S subunits labeled by expression of the ribosomal protein S2-mEos2.

While the static two-state model fits the data reasonably well (Fig. 3A), if it were completely adequate then a value of χ_ν_^2^ as large as 1.24 would be statistically highly unlikely (*P* ~ 0.01). Here we must recognize that the true diffusive behavior of EF-Tu is surely a composite of many diffusive states: free EF-Tu and free ternary complexes (to which the fast diffusion is assigned) and EF-Tu bound to 70S ribosomes and polysomes of variable length (to which the slow diffusion is assigned). Under our fast imaging conditions, the distribution of measured step lengths for the slower population is dominated by the measurement error, not by true displacement of the tracked species. There is also the likelihood of transitions between these states on the 2-ms time scale of the single-step displacement measurements (described below).

What is robust in the fitting results is the fraction of rapidly diffusing copies having a *D*_fast_ value of ~4.9 µm^2^/s. The best-fit fraction *f*_fast_ is 0.40 ± 0.05 in the two-state modeling and 0.35 ± 0.05 in the three-state modeling. Such a fraction of fast molecules is evidently necessary to fit the long tail on the distribution *P*_EF-Tu_(*r*) (Fig. 3A; Fig. S1C), and that is the part of the distribution least perturbed by measurement error. In addition, the *D*_fast_ value of ~4.9 µm^2^/s will be confirmed below in studies of cells treated with the drug rifampin (Rif). The main conclusion of this work—that ~60% of EF-Tu copies are not in the rapidly diffusing states over the 2-ms frame time of the measurements—appears quite robust. In what follows, we proceed with further analysis of the two-state model results under the assumption that they represent the partitioning into ribosome-bound and unbound EF-Tu copies fairly accurately. Separate axial distributions for slow and fast steps (below) will further corroborate the assignments of the fast and slow components.

The best-fit value *D*_slow_ = 1.0 ± 0.2 μm^2^/s for EF-Tu is 10 times larger than the estimated diffusion coefficient of the slow component of the ribosome distribution, *P*_ribo_(*r*), which has a diffusion coefficient of 0.1 ± 0.1 μm^2^/s (Table 1; see Fig. S2 in the supplemental material). Importantly, fits to two-state model functions with the slow diffusion constrained to match that of the 70S ribosomes were much worse (Fig. S1A). This suggests to us that the slow component of EF-Tu diffusion is itself a composite state comprising two substates that exchange with each other during the 2-ms camera frame: EF-Tu bound to 70S ribosomes (with mean lifetime τ_on_) and free EF-Tu or free ternary complexes (with mean lifetime τ_off_) sequestered in the ribosome-rich regions and diffusing freely between ribosome binding events. Here τ_on_ is the mean time a ternary complex spends bound to a 70S ribosome and τ_off_ is the mean time a ternary complex spends searching for a ribosomal binding site, with both times referring to ternary complexes within the ribosome-rich regions. If this is essentially correct, then we can infer (τ_on_ + τ_off_) ≤ 2 ms. If we assume that *D*_fast_ = 4.9 μm^2^/s applies to the free EF-Tu and ternary complex components in the ribosome-rich regions, then the sequestered EF-Tu copies are spending ~80% of the time actually bound to ribosomes and ~20% of the time in transit between ribosome-binding sites. Those are the population fractions that yield the correct weighted average diffusion coefficient: *D*_*slow*_ = 1.0 μm^2^/s = 0.2 × 4.9 μm^2^/s + 0.8 × 0.1 μm^2^/s. The corresponding lifetime ratio is a τ_on_/τ_off_ value of ~4. According to this interpretation, within the ribosome-rich region EF-Tu copies are exchanging between the ribosome-bound and free EF-Tu states so fast that our 2-ms camera frames can only report on the average diffusive behavior of the bound and free states. As discussed below, such short on and off times make good biochemical sense.

10.1128/mBio.02143-17.3 FIG S2  The single-step displacement probability distribution *P*_ribo_(*r*) for 30S ribosomal subunits labeled by the protein S2-Eos2. The best-fit model of two static states is shown. The slow fraction (blue; *f*_slow_ = 0.7, *D*_slow_ = 0.1 µm^2^/s) is assigned to translating 70S ribosomes. The fast fraction (red; *f*_fast_ = 0.3, *D*_fast_ = 1.2 µm^2^/s) is assigned to free 30S subunits. Download FIG S2, TIF file, 7 MB. Copyright © 2018 Mustafi and Weisshaar. 2018 Mustafi and Weisshaar This content is distributed under the terms of the Creative Commons Attribution 4.0 International license.

10.1128/mBio.02143-17.4 FIG S3  The axial distribution of slow and fast single steps for mutant protein EF-Tu^L148A^. Slow and fast steps are based on the same cutoffs used for WT EF-Tu. The axial coordinates are normalized to −0.5 to 0.5. The slow distribution (blue; *r* < 0.1 µm) seems fairly homogeneous, but the fast distribution (red; *r* > 0.2 µm) shows two distinct peaks near the DNA regions. The ribosome axial distribution (black) is also plotted for comparison. Download FIG S3, TIF file, 7 MB. Copyright © 2018 Mustafi and Weisshaar. 2018 Mustafi and Weisshaar This content is distributed under the terms of the Creative Commons Attribution 4.0 International license.

To test the assignment of the slow population to ribosome-bound EF-Tu, we plotted separate axial location distributions for the slowest (step length, *r* < 0.1 μm) and fastest (*r* > 0.2 μm) components of *P*_EF-Tu_(*r*). The arrows in Fig. 3A mark these cutoffs. According to the best two-state model, the slow cutoff includes steps of which ~80% belong to the slow population, while the fast cutoff includes steps of which ~90% belong to the fast population. The location of each step was assigned as the midpoint of the first and second locations, and the axial coordinates were scaled and normalized as before. The results are shown in Fig. 3B in comparison with the total ribosome axial distribution. The three-peaked distribution of slow steps extends into the end caps as the ribosomes do. The distribution of fast steps avoids the ribosome-rich end caps and is perhaps mildly concentrated in the nucleoid regions. These results are consistent with the slow population preferentially residing within the ribosome-rich regions due to transient binding to 70S and the fast population preferentially residing within the nucleoids.

### Effects of rifampin.

To better characterize the diffusive properties of free EF-Tu/ternary complex, we treated exponentially growing cells with 250 μg/ml of the antibiotic rifampin (Rif) for 3 h prior to plating and imaging of EF-Tu–mEos2. Rif halts transcription and thus effectively stops mRNA production (31, 32). On a time scale of 10 min, the existing mRNA is degraded. Lacking mRNA to translate, the 70S polysomes dissociate into free 50S and 30S subunits. We used 792 trajectories that lasted at least 6 steps or longer from 58 cells to plot the spatial distribution of EF-Tu under Rif treatment. The selected cell lengths varied from 3 to 4 µm; after Rif treatment, the distribution of cell lengths shifts toward smaller values. The heat map shows a fairly uniform distribution of EF-Tu along the long axis of the cell, but with the end caps partially excluded (see Fig. S4A in the supplemental material). As shown earlier (31), under Rif treatment the nucleoids expand to fill the cytoplasmic volume fairly homogeneously. The 30S and 50S ribosomal subunits mix with the expanded DNA; they also occupy the cytoplasmic volume fairly uniformly. The EF-Tu distribution is similar.

10.1128/mBio.02143-17.5 FIG S4  (A) The spatial distribution heat map of EF-Tu after Rif treatment is shown for *E. coli* cells 3 to 4 µm in length. Scale is proportional to the counts per pixel. (B) Axial distribution of EF-Tu after Rif treatment (black) along with a simulated homogeneous distribution (red). (C) *P*_EF-Tu_(*r*) distribution of 7,086 steps for EF-Tu after Rif treatment, along with the best-fit parameters for a model of two static states. Download FIG S4, TIF file, 17.1 MB. Copyright © 2018 Mustafi and Weisshaar. 2018 Mustafi and Weisshaar This content is distributed under the terms of the Creative Commons Attribution 4.0 International license.

We used 1,181 trajectories from 78 cells for the EF-Tu diffusive state analysis after Rif treatment. All trajectories of 6 steps or longer were truncated at the sixth step as before. The mean EF-Tu diffusion coefficient obtained from the MSD(τ) plot increases to 3.5 ± 0.4 µm^2^/s (Fig. 2). This is larger than that of EF-Tu in normally growing cells, 2.02 ± 0.09 µm^2^/s. Accordingly, under Rif treatment, the two-state analysis of *P*_EF-Tu_(*r*) (Fig. S4C) finds *f*_slow_ = 0.35 ± 0.05 of EF-Tu that moves with *D*_slow_ = 1.5 ± 0.5 µm^2^/s, slightly larger than the value of *D*_slow_ = 1.0 ± 0.2 μm^2^/s in untreated cells (Fig. 3A). A larger fraction (*f*_fast_ = 0.65 ± 0.05) of EF-Tu moves with the same *D*_*fast*_ = 4.9 ± 1.5 µm^2^/s found for untreated cells. The results after Rif treatment suggest the possibility of some residual binding of EF-Tu/ternary complex to ribosomal subunits, perhaps to the same L7/L12 binding sites on 50S. This is only a suggestion, but it is supported by the results for a mutated variant of EF-Tu presented next.

### EF-Tu^L148A^ mutant.

Rodnina and coworkers (5) studied the effects of point mutations within the C terminus of L7/L12 and within helix D of EF-Tu on the kinetics of initial binding of ternary complex to ribosomes. The mutation sites were chosen by analogy to the well-characterized structure of the EF-Ts/EF-Tu complex. The mutations that caused a substantial decrease in the association rate constant *k*_1_ were used to model the important contacts in the complex between L7/L12 and EF-Tu. The particular mutation L148A in EF-Tu decreased *k*_1_ by a factor of 5. To probe this interaction *in vivo*, we engineered a plasmid containing the same L148A mutation to EF-Tu appended to a C-terminal mEos2 label (Table S1). The mutated protein was expressed in the same background strain, VH1000, along with WT protein expressed normally from the chromosome to enable normal cell growth.

**FIG 4:**
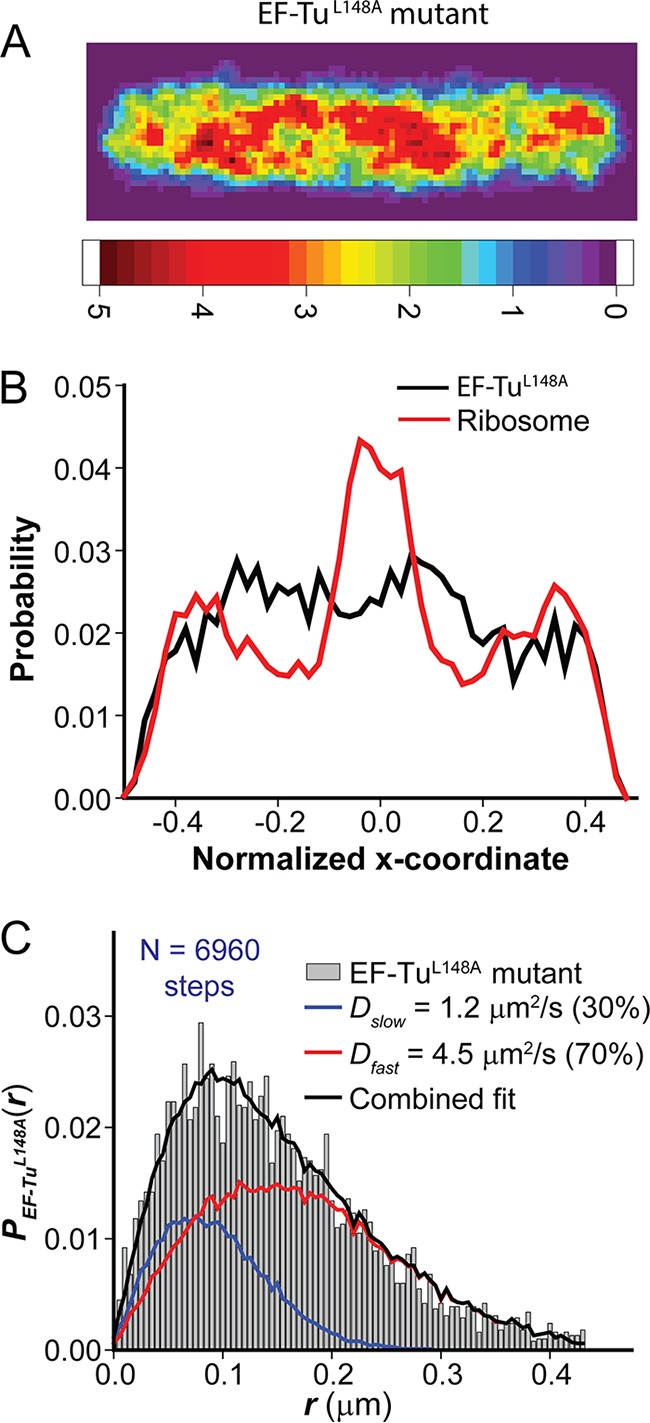
(A) Composite spatial distribution heat map of the mutant form EF-Tu^L148A^–mEos2 for 792 localizations from 123 *E. coli* cells of length 4 to 5.5 µm. Pixels are ~45 by 45 nm. The intensity scale shows relative counts per pixel. (B) Axial distributions of EF-Tu^L148A^ mutant (black) in comparison with ribosomes (30S-mEos2 labeling [red]). The distributions are normalized to the same area and plotted on a relative scale of −0.5 to +0.5 for the long axis. (C) Distribution of single-step displacements *P*(*r*) (gray histogram) for 6,960 steps of the EF-Tu^L148A^ mutant. The solid black line shows the best-fit model using two static states: “slow” (blue) and “fast” (red). Model parameters: *f*_slow_ = 0.3, *D*_slow_ = 1.2 µm^2^/s, *f*_fast_ = 0.7, and *D*_fast_ = 4.5 µm^2^/s.

We obtained 1,160 trajectories of 6 steps or longer from 153 cells to study the diffusion of EF-Tu^L148A^–mEos2. The mean diffusion coefficient from the MSD plot is 3.1 ± 0.3 µm^2^/s (Fig. 2). This is larger than the mean value 2.02 ± 0.19 µm^2^/s for normal EF-Tu–mEos2, consistent with a smaller degree of binding of the mutated protein to ribosomes. Accordingly, the two-component *P*(*r*) analysis of mutant protein diffusion finds *f*_slow_ = 0.30 ± 0.05 (2-fold smaller than for the normal protein) with *D*_slow_ = 1.2 ± 0.5 µm^2^/s and *f*_fast_ = 0.70 ± 0.05 with *D*_fast_ = 4.5 ± 1.0 µm^2^/s (Fig. 4C). The location heat map and the axial spatial distribution for the EF-Tu^L148A^ mutant (Fig. 4A and B) show that the mutated protein is fairly uniformly distributed throughout the cell, with only a hint of three peaks. These results indicate substantially less binding of the EF-Tu^L148A^ mutant to ribosomal sites, in qualitative agreement with the mutation studies *in vitro* (5). The agreement helps to corroborate our underlying assumption that ternary complexes are binding to L7/L12 ribosomal subunits *in vivo*; see Discussion for a summary of additional biochemical evidence.

To control for possible effects of overexpression of the L148A mutant from the plasmid, we constructed an analogous plasmid that expresses WT EF-Tu–mEos2 and incorporated it into the same VH1000 background strain. The spatial distribution and diffusive properties of the EF-Tu–mEos2 copies expressed from the plasmid were qualitatively similar to those of EF-Tu–mEos2 expressed from the chromosome (see Fig. S5 in the supplemental material).

10.1128/mBio.02143-17.6 FIG S5  Single-step displacement probability distribution *P*_EF-Tu_(*r*) for EF-Tu expressed from a plasmid. The best-fit two-state model parameters are as follows: blue curve, *f*_slow_ = 0.7 and *D*_slow_ = 1.3 µm^2^/s; red curve, *f*_fast_ = 0.3 and *D*_*fast*_ = 4.9 µm^2^/s. The diffusion of WT EF-Tu expressed from a plasmid is similar to the diffusion of WT EF-Tu expressed from the chromosome (Fig. 3). Download FIG S5, TIF file, 7.2 MB. Copyright © 2018 Mustafi and Weisshaar. 2018 Mustafi and Weisshaar This content is distributed under the terms of the Creative Commons Attribution 4.0 International license.

10.1128/mBio.02143-17.7 FIG S6  (A) Simulated distributions of the mean of six single-step estimates of the diffusion coefficient, *P*_model_(*D*_*i*_), for two diffusion coefficients, *D*_slow_ and *D*_fast_, as indicated. These distributions were used to estimate cutoff values for slow and fast copies of EF-Tu, whose experimental distribution is shown in panel (B). (C) Separate MSD plots for slow and fast EF-Tu copies. Intercepts were used to set different values of localization error: σ_slow_ = 40 nm and σ_fast_ = 80 nm. Download FIG S6, TIF file, 16.3 MB. Copyright © 2018 Mustafi and Weisshaar. 2018 Mustafi and Weisshaar This content is distributed under the terms of the Creative Commons Attribution 4.0 International license.

10.1128/mBio.02143-17.8 FIG S7  Planes passing through the three-dimensional grid search for the best χ_ν_^2^ value for the two-state fits to WT EF-Tu distribution *P*_EF-Tu_(*r*) of Fig. 3. Each slice shows a plane passing through the global minimum parameter set: (A) *f*_slow_ is fixed at 0.60. (B) *D*_slow_ is fixed at 1.0 µm^2^/s. (C) *D*_fast_ is fixed at 4.9 µm^2^/s. The uncertainty estimate for each parameter was chosen to enclose all values of χ_ν_^2^ within 0.5 U of the minimum value, as shown by the boxed-in regions of each plane. Fits with still larger values of χ_ν_^2^ were judged to be qualitatively poor. Download FIG S7, TIF file, 23.5 MB. Copyright © 2018 Mustafi and Weisshaar. 2018 Mustafi and Weisshaar This content is distributed under the terms of the Creative Commons Attribution 4.0 International license.

To test for possible binding of the mutant form EF-Tu^L148A^ to free 50S ribosomal subunits, we obtained 993 trajectories of 6 steps or longer from 83 cells after the 3-h Rif treatment. The slope of the MSD plot increases to 5.2 ± 0.4 μm^2^/s (Fig. 2), compared with 3.5 ± 0.4 µm^2^/s for WT EF-Tu after Rif. The *P*(*r*) distribution is fit qualitatively by a single population with *D* = 5.7 ± 1.0 μm^2^/s (χ_ν_^2^ = 1.5). The best two-component fit yielded *f*_slow_ = 0.10 ± 0.05, *D*_slow_ = 1.9 ± 1.2 μm^2^/s, *f*_fast_ = 0.90 ± 0.05, *D*_fast_ = 5.6 ± 1.2 μm^2^/s, and χ_ν_^2^ = 1.2. The analysis indicates that after Rif treatment, most EF-Tu^L148A^ is diffusing essentially freely, perhaps primarily as ternary complexes. Evidently the mutant protein exhibits little or no binding to free 30S or 50S subunits.

### Numerical estimates and comparisons with theory.

The present results can be combined with literature estimates for relative copy numbers of ribosomes, EF-Tu, EF-G, tRNAs, and aa-tRNA synthetases to provide semiquantitative insight into the partitioning of EF-Tu and tRNA across functional states and the time-averaged stoichiometry of the species bound to a translating ribosome. Under the same growth conditions used here (30°C in EZRDM), we previously estimated ~50,000 30S ribosomal subunits per cell, some 80% of which (~40,000 copies) are engaged as translating 70S ribosomes (15). Mean copy number estimates for EF-Tu, total tRNA, EF-G, and total aa-tRNA synthetase (Table S1) were derived from the ribosome copy number and from literature values of the ratio of each species’ copy number to that of ribosomes. It was not possible to match strains, growth conditions, growth rates, and temperatures, so we chose to match only the growth rate (~1 doubling/h). We hope these rough estimates will help constrain future models of overall *E. coli* translation rates. Their biological significance will be discussed further below. Details of the calculations and underlying assumptions are provided in the supplemental material; here we summarize the estimates. The primary assumption is that EF-Tu binds to translating ribosomes via contact with the C terminus of L7/L12. This is justified in the Discussion.

The time-averaged stoichiometry of EF-Tu and tRNA binding to a translating 70S ribosome can be estimated from the fraction of EF-Tu copies bound to ribosomes combined with copy number estimates from other studies. There are 61 different codons and 43 different aa-tRNA types (43 different ternary complexes) used by *E. coli* (9). Forty-eight codons match only one type of ternary complex, 12 match two types, and one matches three types. This means that the ribosome is usually testing and rejecting noncognate or near-cognate aa-tRNAs. The A site is most frequently occupied by an aa-tRNA within its ternary complex, still tethered to L7/L12 (prior to codon selection, GTP hydrolysis, and ejection of EF-Tu) (9).

Under our growth conditions of 30°C in EZRDM, we estimate the following mean copy numbers per cell: ~40,000 translating 70S ribosomes (concentrated in three ribosome-rich regions) (15), ~10,000 free 30S subunits, ~10,000 free 50S subunits, ~350,000 total EF-Tu copies (10, 33), ~350,000 total tRNA copies (34), ~50,000 EF-G copies (which compete with EF-Tu for L7/L12 binding sites) (33), and ~50,000 aa-tRNA synthetases (33). The new data suggest that ~210,000 EF-Tu copies (60% [the “slow” copies]) are ternary complexes that occupy the ribosome-rich regions, where they are bound to 70S ribosomes ~80% of the time (~170,000 ribosome-bound ternary complexes plus ~40,000 free ternary complexes). Thus, we estimate as many as ~170,000/40,000 = 4 ternary complexes bound to each translating ribosome. This indicates that the four L7/L12 subunits in *E. coli* are essentially saturated with ternary complexes. However, EF-G must also bind to L7/L12 in order to drive translocation on those rare occasions when a cognate aa-tRNA is accommodated in the A site and forms a new peptide bond. In our estimate, we assume the average occupancy of the four L7/L12 units is 3.5 ternary complexes and 0.5 EF-G copy. The remaining ~180,000 EF-Tu copies partition into ~70,000 free ternary complexes plus ~110,000 free (bare) EF-Tu copies. The overall partitioning of EF-Tu between ternary complexes and free EF-Tu is corroborated by an equilibrium calculation based on the aa-tRNA/EF-Tu binding constant *in vitro* (35).

For partitioning of the ~350,000 total tRNA copies, we estimate that on average each 70S ribosome binds one tRNA at the A site (usually tethered to L7/L12 by a bridging EF-Tu), one in the P site, one-half tRNA in the E site (an average over “2-1-2” and “2-3-2” models [36, 37]), plus an additional ~2.5 tRNAs bound to the other three L7/L12 sites. Recall that EF-G is assumed to take up 0.5 L7/L12 binding site. Thus, averaged over time, ~5 tRNAs are bound to each 70S ribosome (~200,000 tRNAs bound to ~40,000 translating ribosomes, comprising ~140,000 tRNAs within ternary complexes and ~60,000 tRNAs at the P and E sites). The remaining ~150,000 tRNA copies not bound to 70S are estimated to partition among three states: ~50,000 copies being recharged by aa-tRNA synthetases, ~100,000 copies within free ternary complexes, and only ~400 free tRNAs. These estimates are based in part on an equilibrium calculation using the *in vitro* binding constant of aa-tRNA with EF-Tu (35).

In addition, our new data are in sensible agreement with two rate constants from a model that optimally scaled a detailed set of *in vitro* rate constants to derive a set of theoretical *in vivo* rate constants describing the multistep process of the elongation cycle (9). Again, details are presented in the supplemental material. First we use the pseudo-first-order rate τ_off_^−1^ and the 70S ribosome concentration to estimate a lower limit on the effective bimolecular association rate constant *k*_1_ for binding of a typical noncognate ternary complex to an L7/L12 subunit of a 70S ribosome within the ribosome-rich regions. The result is *k*_1_ = τ_off_^−1^/[70S] ≥ 4.5 × 10^7^ M^−1^ s^−1^. This is remarkably fast, at least 1/6 of the calculated diffusion-limited rate constant *k*_diff_ = 3.2 × 10^8^ M^−1^ s^−1^. As suggested earlier (4), *k*_1_ (which is expressed on a per ribosome basis) may be especially large due to the four L7/L12 binding sites per ribosome and the length and flexibility of the linkages between ribosome and the C-terminal domain of L7/L12. The theoretical *in vivo* estimate for the analogous κ_on_* (see Table 2 in reference 9) at 1.07 doublings/h and 37°C is 9.4 × 10^7^ M^−1^ s^−1^, 2-fold larger than our lower limit on *k*_1_.

We can also compute a lower limit on the unimolecular dissociation rate of EF-Tu (usually as part of a ternary complex) from the ribosome, *k*_−1_ = τ_on_^−1^ ≥ 625 s^−1^ at 30°C. The value of *k*_*−*1_ is temperature sensitive. If we apply an Arrhenius-based correction factor of 2.1 to our *k*_*−*1_ value at 30°C (details in supplemental material), the estimated value at 37°C becomes *k*_−1_ ≥ 1,250 s^−1^. This is consistent with the theoretical *in vivo* rate constant for 1.07 doublings/h at 37°C, ω_off_* = 1,700 s^−1^ (see Table 2 in reference 9).

### Disagreement with a recent tRNA tracking study.

In violation of the standard model of aa-tRNA recruitment, a recent single-tRNA tracking experiment from the Kapanidis lab inferred that a large majority of tRNA copies exist as free tRNA, bound neither to EF-Tu in ternary complexes nor to the aminoacyl-tRNA synthetase (20). They electroporated a small number of tRNA copies fluorescently labeled with Cy5 dye into *E. coli* and tracked the motion of single molecules. A large fraction (70 to 90%) of the tRNA-Cy5 copies diffused very rapidly (corrected *D*_tRNA_ value of ~8 μm^2^/s). These copies were attributed to free tRNA (not bound within ternary complexes). The conclusion was that diffusion of free aa-tRNA, not ternary complexes, must be the primary means of delivery of aa-tRNA to the ribosomal A site. The remarkably large fraction of free tRNA copies was deemed possible based on the assumption that only two tRNA copies are bound to each ribosome (one each in the A and P sites). The rationale given for the small estimated fraction of ternary complexes (20) was that EF-Tu can bind to membrane-bound MreB, as evidently occurs in both *Bacillus subtilis* and *E. coli* (38–40). This would remove EF-Tu from the cytoplasm and make it less available for ternary complex formation. However, the EF-Tu copy number is about 100 times larger than that of MreB (33). In addition, we find no evidence in our EF-Tu spatial distribution of significant binding to the cytoplasmic membrane, where MreB resides. In contrast, our numerical estimates based on an average of ~3.5 ternary complexes bound to the four L7/L12 sites indicate ~5 bound tRNA copies per ribosome. Finally, our equilibrium calculations suggest that only ~1% or less of total tRNA should exist as free tRNA.

One potential weakness of the electroporation method (20) is that the few labeled tRNA copies in each cell must compete with the 350,000 endogenous tRNA copies for aminoacylation, ternary complex formation, and binding and processing by the ribosome. Although the labeled tRNA-Cy5 species was shown to be functional *in vitro*, it is difficult to know how well tRNA-Cy5 copies compete with endogenous copies in each functional step *in vivo*. It seems possible that the synthetase recognizes tRNA-Cy5 poorly, aa-tRNA–Cy5 forms ternary complexes poorly *in vivo* or these complexes bind 70S ribosomes weakly, or Cy5 fluorescence is somehow quenched in ternary complexes so that they are not detected.

## DISCUSSION

### Rapid testing of aa-tRNA copies for a codon-anticodon match.

In rapidly growing *E. coli*, the mean protein elongation rate can be as fast as 20 amino acids/s. Single elongation cycles must be carried out in less than ~50 ms (7). There are 61 different codons and 43 different aa-tRNA types (43 different ternary complexes) (9). Forty-eight codons match only one type of ternary complex, 12 match two types, and 1 matches three types. Fully 40 unique codons are used with at least 1% frequency (41). For a given mRNA codon poised at the 30S decoding site, the average chance that a particular ternary complex carries a cognate (completely matching) aa-tRNA anticodon is roughly 1 in 40. This means on average, approximately 40 different ternary complexes must be sampled before a cognate aa-tRNA is found. (See the supplemental material for the probabilistic calculation.) Sampling and testing of these complexes must occur faster than the complete elongation cycle time of 50 ms, suggesting an upper limit of ~1 ms on the average time taken for ternary complex evaluation.

Selection for cognate aa-tRNA is a two-stage process (1, 9). Essentially all noncognate ternary complexes and a large majority of near-cognate ternary complexes dissociate from L7/L12 in the initial recognition stage, prior to GTP hydrolysis by EF-Tu. This can be seen from the “theoretical *in vivo*” rate constants of Lipowsky and coworkers (9). Those events should dominate our single-molecule observations. The small fraction of near-cognate ternary complexes that pass through the initial stage is efficiently rejected in the proofreading stage, which occurs after GTP hydrolysis (9). Only cognate aa-tRNAs move forward rapidly through both stages, efficiently achieving A state accommodation.

Our single-molecule tracking study provides some new insight into the spatial distribution and time scale of binding and unbinding events between EF-Tu (ternary complexes) and translating ribosomes in *E. coli*. These methods cannot dissect binding events for cognate versus near-cognate versus noncognate ternary complexes. Instead, the measurements probe the time scale of the initial, codon-independent binding and unbinding with L7/L12. The new *in vivo* results corroborate several mechanistic inferences previously gleaned from a large body of *in vitro* kinetics measurements (1). Evidently the high concentration of ternary complexes, the segregation of 70S ribosomes in the ribosome-rich regions of the cytoplasm, the presence of four L7/L12 binding sites per 70S ribosome, and the flexible attachment of the L7/L12 binding sites to the ribosome all combine to enable extremely rapid sampling of aa-tRNA copies by the 70S ribosome.

Our interpretation of *D*_slow_ = 1 μm^2^/s as arising from a composite state involving rapid exchange between 80% ribosome-bound ternary complexes (τ_on_) and 20% free ternary complexes (τ_off_) within the ribosome-rich regions led to the inequality (τ_on_ + τ_off_) ≤ 2 ms. This result is consistent with the requisite fast sampling and rejection of ternary complexes required by the predominance of noncognate and near-cognate aa-tRNAs. The estimated lower bounds on the bimolecular binding rate constant *k*_1_ and the unimolecular dissocation rate *k*_−1_ are consistent with recent theoretical estimates of the analogous in vivo rate constants (9). The novel method used for scaling of *in vitro* rates to find the optimal set of *in vivo* rates that match the overall *E. coli* translation rate seems remarkably successful.

### Ribosomal L7/L12 sites bind multiple ternary complexes simultaneously.

The new data provide strong evidence that multiple ternary complexes bind simultaneously to the four L7/L12 sites on the 50S subunit of translating ribosomes. Our partitioning analysis suggests that the four L7/L12 sites may be saturated with ternary complexes on average. Such a high local concentration of tethered aa-tRNAs would greatly facilitate the rapid sampling required for efficient protein elongation, as previously suggested (4). The enhanced sampling rate would arise from two effects. During the same time interval in which one of the bound ternary complexes is being tested, any open L7/L12 site can be replenished with a fresh ternary complex. This saves time. In addition, when an A site comes open after a codon match and translocation or (more typically) after rejection of a noncognate aa-tRNA, the diffusive search for the open A site by a new ternary complex would be more rapid due to the high local concentration and the spatial constraints imposed by the tethering.

There is extensive biochemical evidence *in vitro* supporting our underlying assumption that aa-tRNA–EF-Tu(GTP) ternary complexes bind the ribosome via contact between L7/L12 and EF-Tu. A comprehensive summary is provided in reference 4. As shown schematically in Fig. 1A, L7/L12 comprises an N-terminal dimerization module and a globular C-terminal domain (CTD) connected by a flexible hinge. In *E. coli*, four copies of L7/L12 are bound to L10, which is itself flexible. An early chemical cross-linking and fluorescence study implicated L7/L12 in the binding of EF-Tu to the ribosome (6). Subsequent extraction/complementation experiments showed that the presence of L7/L12 was required for binding of both EF-Tu and EF-G to the ribosome (42). Specific point mutations in the L7/L12 CTD and in the G domain of EF-Tu affected binding of ternary complexes to the ribosome (5). In addition, there is homology between the proposed L7/L12 binding interface to EF-Tu and the well-characterized structure of the EF-Ts/EF-Tu complex. The L7/L12 subunits do not appear in crystal structures of 70S ribosomes (2, 43). However, the biochemical evidence is corroborated by an early reconstruction from cryo-EM data with a 1.8-nm resolution that shows density connecting the G domain of EF-Tu within a ternary complex to the L7/L12 stalk of the ribosome (13). Finally, the correspondence between the diminished binding of the mutant form EF-Tu^L148A^ *in vitro* (5) and in live *E. coli* cells (Fig. 4) corroborates the assertion that we are probing ternary complex binding to L7/L12.

Wahl and coworkers (4) combined biochemical and additional structural evidence to propose the model of the stalk that we reproduce schematically in Fig. 1A. The schematic shows four ternary complexes bound to the ribosome via the four L7/L12 CTDs. One ternary complex is undergoing codon testing at the A site, while the other three are tethered and awaiting testing. The flexible attachment of the four L7/L12 CTDs to the ribosome is likely to facilitate efficient capture of ternary complexes. The long, flexible linkers may enable the CTDs to “reach out and catch” ternary complexes that come into near proximity of the ribosome body (4, 44). Although there is no detailed structural evidence supporting the simultaneous binding of four ternary complexes, the concept is supported by our stoichiometric estimates in vivo. This concept is also supported by the remarkably large bimolecular rate constant for ternary complex binding to 70S, measured earlier *in vitro* and now estimated *in vivo*.

### Conclusions.

The present work provides strong evidence that multiple ternary complexes bind the four L7/L12 initial binding sites on the 50S subunit of the 70S ribosome simultaneously. We also provide a new estimate of ~1 to 2 ms or less for the *in vivo* time scale of binding and unbinding of noncognate ternary complexes during the initial anticodon test. Semiquantitative estimates of the partitioning of EF-Tu and tRNA among different binding states should help constrain models of translation in *E. coli*. In future work, tracking studies of EF-G could provide an independent estimate of the fraction of EF-G bound to 70S ribosomes at a given moment in time. That would shed light on the competition *in vivo* between EF-Tu and EF-G for L7/L12 binding sites on the 70S ribosome.

## MATERIALS AND METHODS

### Bacterial strains.

We chose 30°C for this study because the mEos2 labels fluoresce poorly at 37°C; also, 30°C matches the conditions of our earlier study of ribosome copy number, a result used here (19). The strains, doubling times, and oligonucleotides used are detailed in Table S1. In *E. coli*, EF-Tu is expressed from two essentially identical genes: *tufA* and *tufB*. Both of these genes were first labeled endogenously via the lambda red technique (45) in the background strain, NCM3722. The photoconvertible fluorescent protein mEos2 was covalently bound to the C terminus of EF-Tu. These genes were then transferred to the VH1000 background strain using P1 transduction. For studies of the mutated protein EF-Tu^L148A^, the *tufA* gene was point mutated from Leu to Ala at the 148th residue in a plasmid with ampicillin resistance, pASK-IBA3+. The plasmid mutation included a fusion of the same mEos2, again at the C terminus of the protein. To control for possible effects of overexpression, we also prepared a strain including a completely analogous plasmid, except that it lacked the point mutation. The 30S ribosomal subunits were labeled by expression of the protein S2-mEos2 from the chromosome.

In “EZ rich, defined medium” (EZRDM) at 30°C, the doubling time of the endogenously labeled *tufA* and *tufB* strain is 60 ± 3 min (Table S1A; Fig. S8). This is ~1.3 times longer than the doubling time of the VH1000 background strain, which is 45 ± 2 min, indicating that the mEos2 label enables fairly normal functionality of EF-Tu. The L148A mutant strain has a doubling time of 46 ± 4 min.

10.1128/mBio.02143-17.9 FIG S8  (A) Growth curves in EZRDM at 30°C for three different strains: VH1000 (WT parent strain, no labeling), MSG195 (VH1000 with ribosomal protein S2 labeled with mEos2), and tufAB (*tufA* and *tufB* both labeled with mEos2). The exponential-phase data points are used to calculate the doubling times. (B and C) Plots of ln OD/ln2 versus time for the VH1000 and tufAB strains, respectively. The exponential-phase data points are fitted to a straight line whose inverse slope yields doubling times of 45 ± 2 min for VH1000 and 60 ± 3 min for tufAB. Download FIG S8, TIF file, 10.5 MB. Copyright © 2018 Mustafi and Weisshaar. 2018 Mustafi and Weisshaar This content is distributed under the terms of the Creative Commons Attribution 4.0 International license.

### Cell growth and preparation for imaging.

Bulk cultures from frozen glycerol stock solution and subcultures for imaging were grown overnight at 30°C with continuous shaking in EZRDM, which is a morpholinepropanesulfonic acid (MOPS)-buffered solution with supplemental metal ions (M2130; Teknova), glucose (2 mg/ml), supplemental amino acids and vitamins (M2104; Teknova), nitrogenous bases (M2103; Teknova), 1.32 mM K_2_HPO_4_, and 76 mM NaCl. The next day, the stationary-phase culture was diluted 100-fold in fresh EZRDM and grown again to exponential phase (optical density [OD] of 0.2 to 0.5). Cells were then plated on a polylysine-coated coverslip that formed the floor of a CoverWell perfusion chamber (Invitrogen, Carlsbad, CA) with a well volume of 140 µl.

For the L148A mutant strain, when the culture reached exponential phase it was treated with anhydrous tetracycline (final concentration, 45 nM) to induce expression of EF-Tu^L148A^–mEos2 from the plasmid. Tetracycline was washed away after 5 min of induction, and the cells were grown for 30 min more in fresh medium prior to plating and imaging. To test for the effects of treatment by rifampin (Rif), cells were grown to the exponential phase, after which Rif was added to a final concentration of 250 µg/ml. The culture remained at 30°C for 3 h, after which cells were plated and imaged.

### Superresolution imaging of live *E. coli* cells.

Imaging of cells began within 5 min of plating. Individual fields of view were imaged no longer than 20 s to minimize laser damage. Each prepared sample was imaged for no longer than 30 min, during which time cells continued to grow normally. Cells were imaged on an inverted microscope (model Eclipse-Ti; Nikon Instruments, Melville, NY) equipped with an oil immersion objective (CFI Plan Apo Lambda DM 100× oil, 1.45 NA; Nikon Instruments), a 1.5× tube lens, and the Perfect Focus system (Nikon Instruments, Melville, NY). The fluorescence images were recorded on a back-plane illuminated electron-multiplying charge-coupled device (EMCCD) camera (iXon DV-887; Andor Technology, South Windsor, CT) at the rate of 485 Hz (~2 ms/frame). The camera chip consisted of 128 by 128 pixels (px), each 24 by 24 µm. The fluorescent protein mEos2 was activated using a 405-nm laser (CW laser; CrystalLaser, Reno, NV); the photoswitched state was subsequently excited with a 561-nm laser (Sapphire CW laser; Coherent, Inc., Bloomingfield, CT). Both lasers illuminated the sample for the entire duration of image acquisition. Emission was collected through a 617/73 bandpass filter (bright line 617/73-25; Semrock, Rochester, NY). The 405-nm power density at the sample was ~5 to 10 W/cm^2^, which kept the number of activated molecules at less than two in each camera frame. The 561-nm laser power density at the sample was ~8 kW/cm^2^.

### Single-molecule image analysis.

The fluorescent images were analyzed using a MATLAB graphical user interface (GUI) developed in our lab (23). Noise was attenuated using 2 different digital filters. After filtering, fluorescent signals were identified using a peak-finding algorithm with a user-defined intensity threshold with pixel-level accuracy. A particle is identified if the local intensity maximum is higher than the threshold. The threshold is carefully chosen to be large enough so that the algorithm can distinguish between background and signal and small enough to avoid cutting trajectories unduly short.

A centroid algorithm was used to locate the identified particles with subpixel resolution (23). Rapidly moving molecules have images that are blurred asymmetrically due to diffusion from the camera frame. Centroid fitting can locate the particles with better accuracy than Gaussian fitting. The centroid algorithm is also faster computationally. A 7- by 7-px box was drawn around the intensity maxima, and the centroid of all the pixel intensities within the box was calculated. The centroid positions from successive frames were connected to form a trajectory only if they lie within 3 px = 480 nm of each other. A modiﬁed MATLAB version of the tracking program written by Crocker and Grier (46) was used.

### Analysis of diffusive behavior.

Details of spatial distribution, mean-square displacement plots, trajectory simulations, two-state modeling of *P*(*r*) distributions, and estimation of uncertainties in fitting parameters are provided in the supplemental material.

10.1128/mBio.02143-17.1 TEXT S1  The text file describes the data analysis and fitting procedures in detail. It also describes the probabilistic argument for the number of trials required to find an aa-tRNA whose anticodon matches the current mRNA codon and provides a detailed description of the four-ternary-complex binding model. Download TEXT S1, PDF file, 0.3 MB. Copyright © 2018 Mustafi and Weisshaar. 2018 Mustafi and Weisshaar This content is distributed under the terms of the Creative Commons Attribution 4.0 International license.
