# Supplementary material for: Simultaneous Binding of Multiple EF-Tu Copies to Translating Ribosomes in Live Escherichia coli
Source: mBio. 2018 Jan 16;9(1):e02143-17. doi: 10.1128/mBio.02143-17 (PMC5770553; doi:10.1128/mBio.02143-17)
Supplement: TABLE S1 [file mbo001183666st1.pdf]

Table S1A. Bacterial strains used; background strain is *E. coli* – VH1000.

Strain label	Description	Doubling time (min)
tufAB	Both *tufA* and *tufB* chromosomally labeled with mEos2 at C-terminus	60 ± 3
MM7	*tufA* labeled with mEos2, expressed from a plasmid – pASK-IBA3+	47 ± 1
MM9	$tufA^{L148A}$ mutant labeled with mEos2, expressed from a plasmid – pASK-IBA3+	46 ± 4
MSG196	*rpsB* (ribosome S2 protein) chromosomally labeled with mEos2	49 ± 2
VH1000	Background parent strain	45 ± 2

Table S1B. Oligonucleotides used.

Oligonucleotide	Sequence, 5′- 3′
F-EcoRI-tufA_mEos2	**GCAT *GAATTC* CAGGCCGTAATTGAAGC**
R-HinDIII-tufA-mEos2	**GCAT *AAGCTT* TTAGCGACGAGCATTATCC**
F-tufA_L148A_mut	**GCTGCTGGAA *GCA* GTTGAAATGGAAG**
R-tufA_L148A_mut	**TCTTCGTCATCAACCATG**

Table S1C. Sources of copy number estimates relative to ribosomes.[1]

Measured species (references)	Ratio to ribosome copy number	*E. coli* strain	Growth conditions
**total EF-Tu (12, 23)**	7	NC3	MOPS with 0.4% glucose (w/v) at 37˚C (~1 db/hr)
**total tRNA (17)**	7	W1845 (K12 derivative)	MOPS with 0.4% glucose (w/v) at 37˚C (~1 db/hr)
**EF-G (23)**	~1	MG1655	M9 with glucose at 37˚C
**total tRNA synthetase (23)**	~1	MG1655	M9 with glucose at 37˚C

[1] Estimates of mean copy number per cell in main text are taken as the mean ribosome copy number of 50,000 (from Ref. 11 below) times the ratio in this table.
