# Supplementary figures and images for: Simultaneous Binding of Multiple EF-Tu Copies to Translating Ribosomes in Live Escherichia coli
Source: mBio. 2018 Jan 16;9(1):e02143-17. doi: 10.1128/mBio.02143-17 (PMC5770553; doi:10.1128/mBio.02143-17)

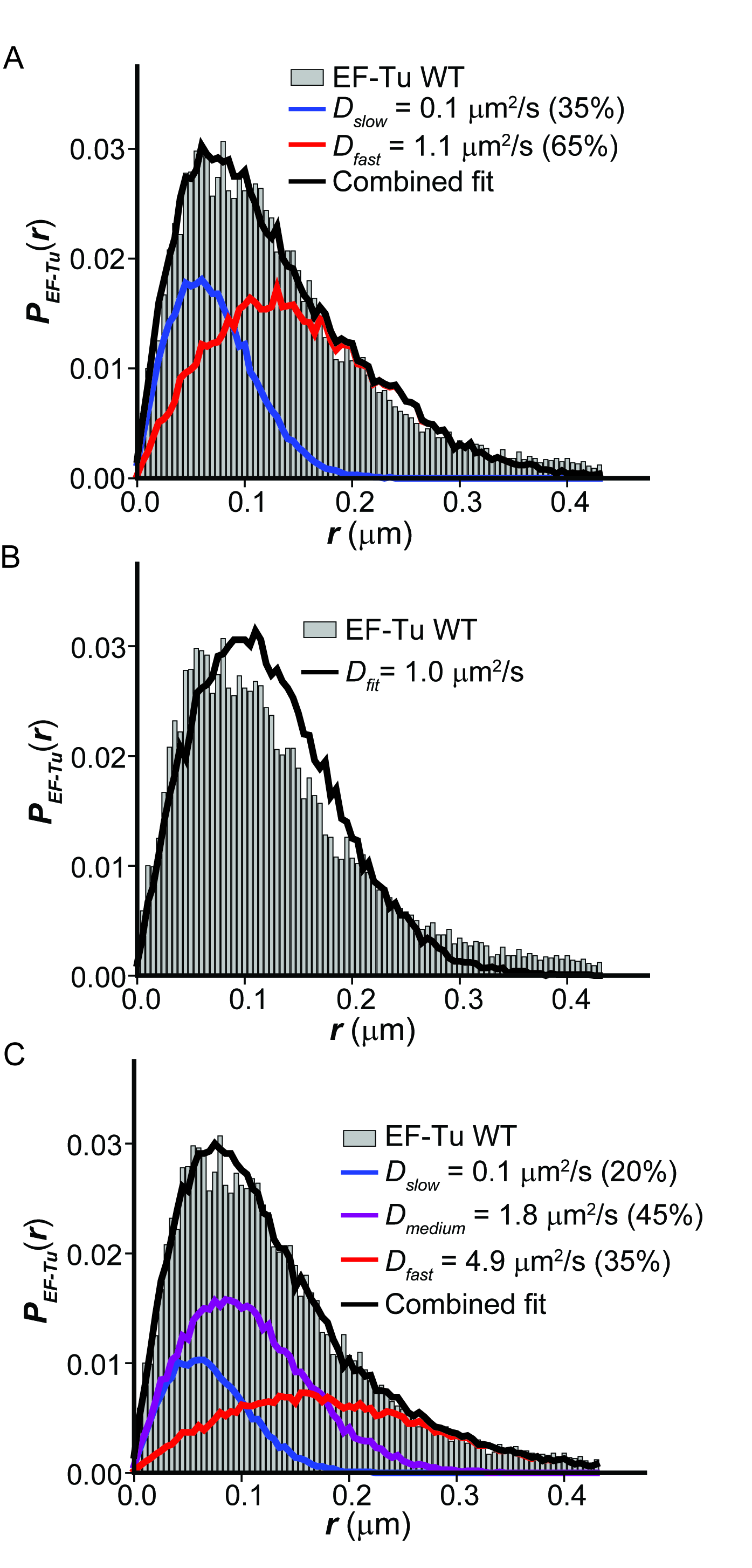

Supplement: FIG S1 [file mbo001183666sf1.tif]

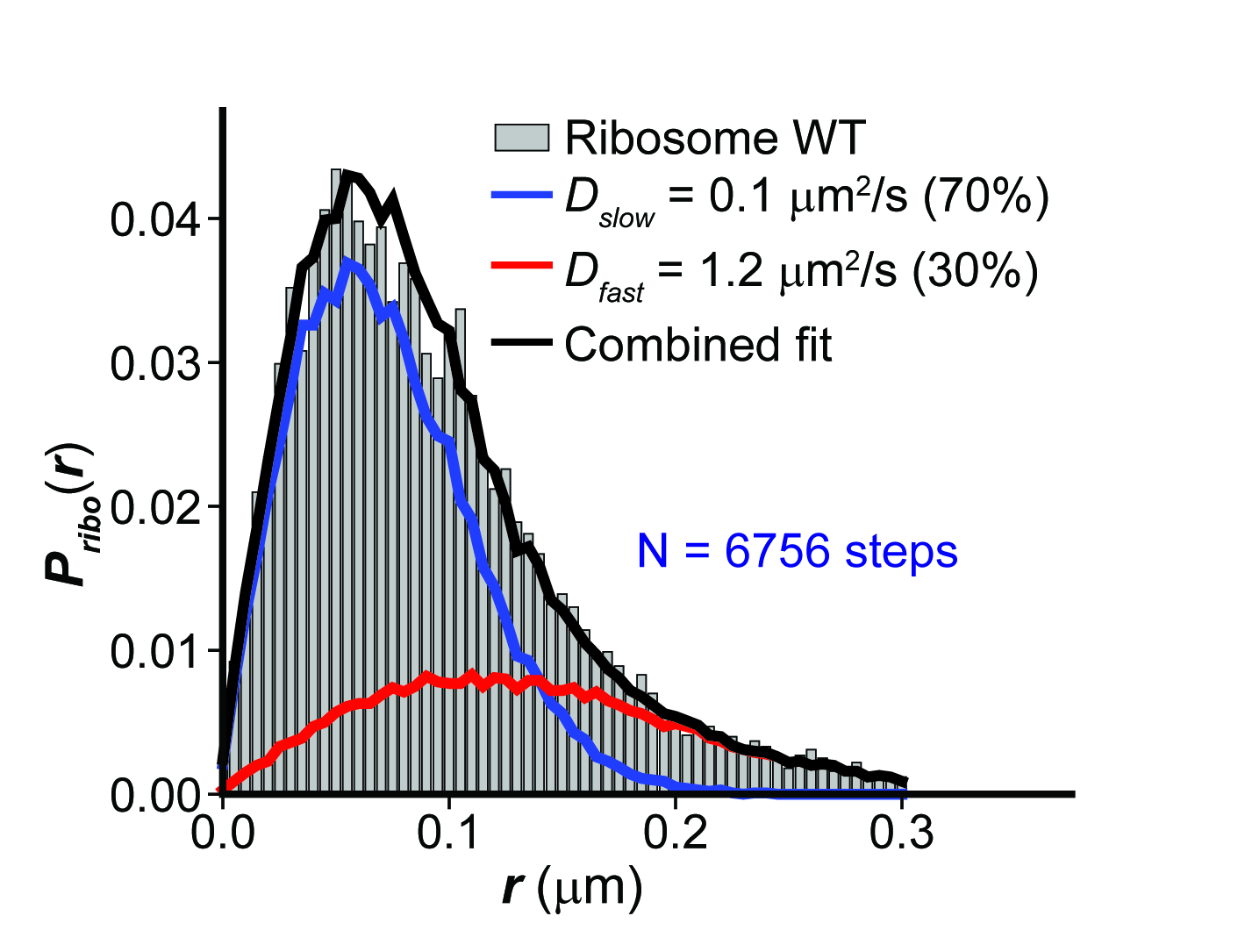

Supplement: FIG S2 [file mbo001183666sf2.tif]

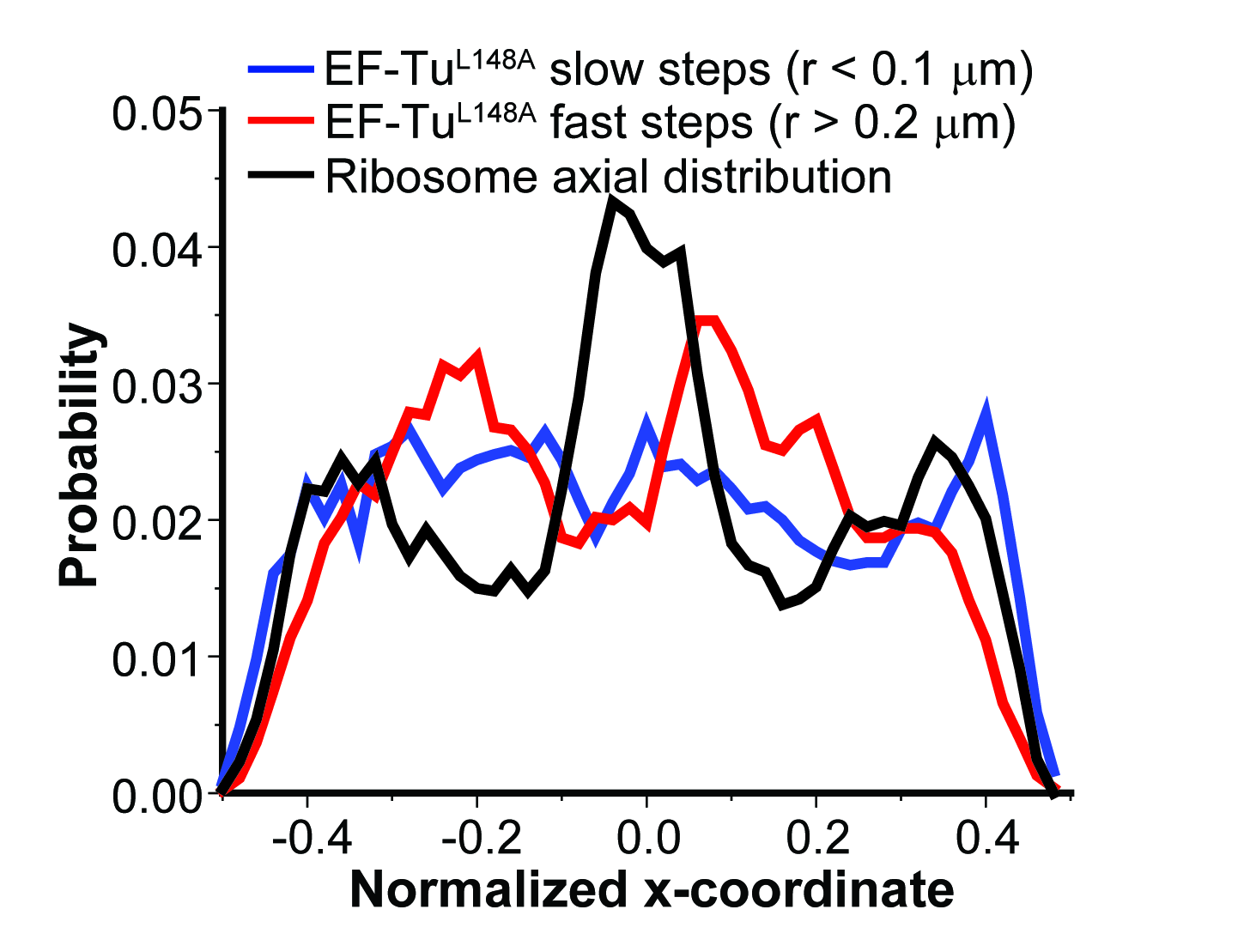

Supplement: FIG S3 [file mbo001183666sf3.tif]

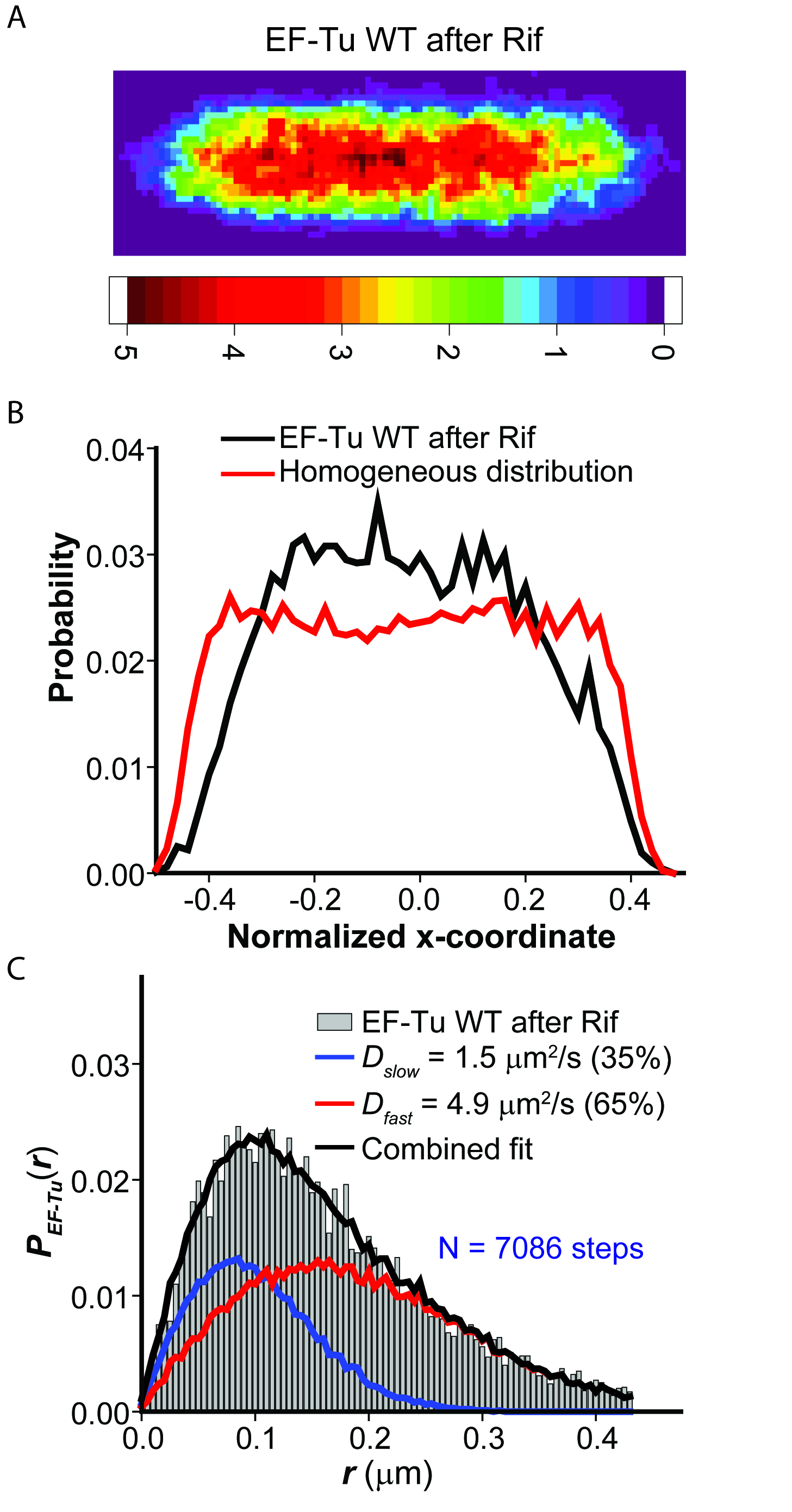

Supplement: FIG S4 [file mbo001183666sf4.tif]

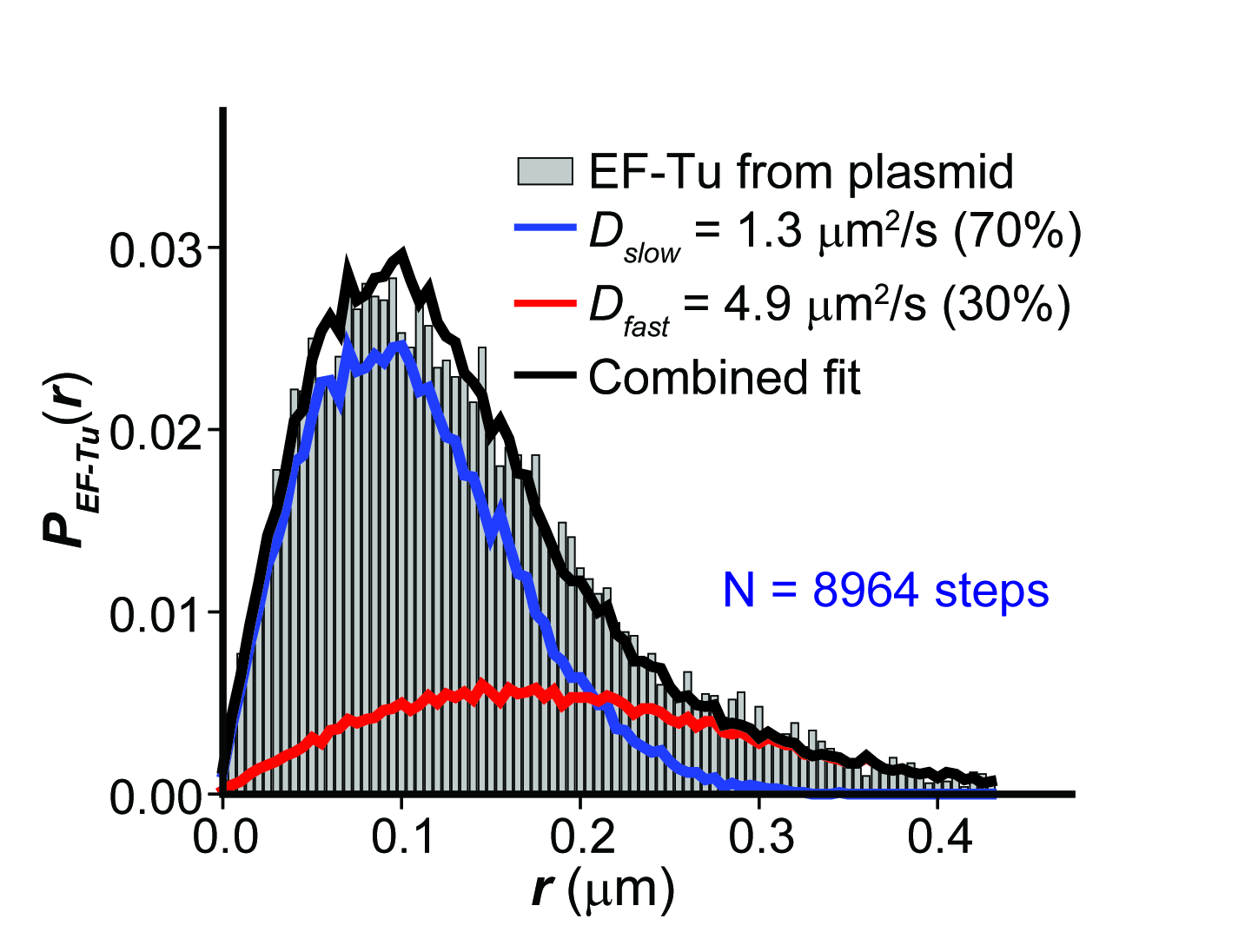

Supplement: FIG S5 [file mbo001183666sf5.tif]

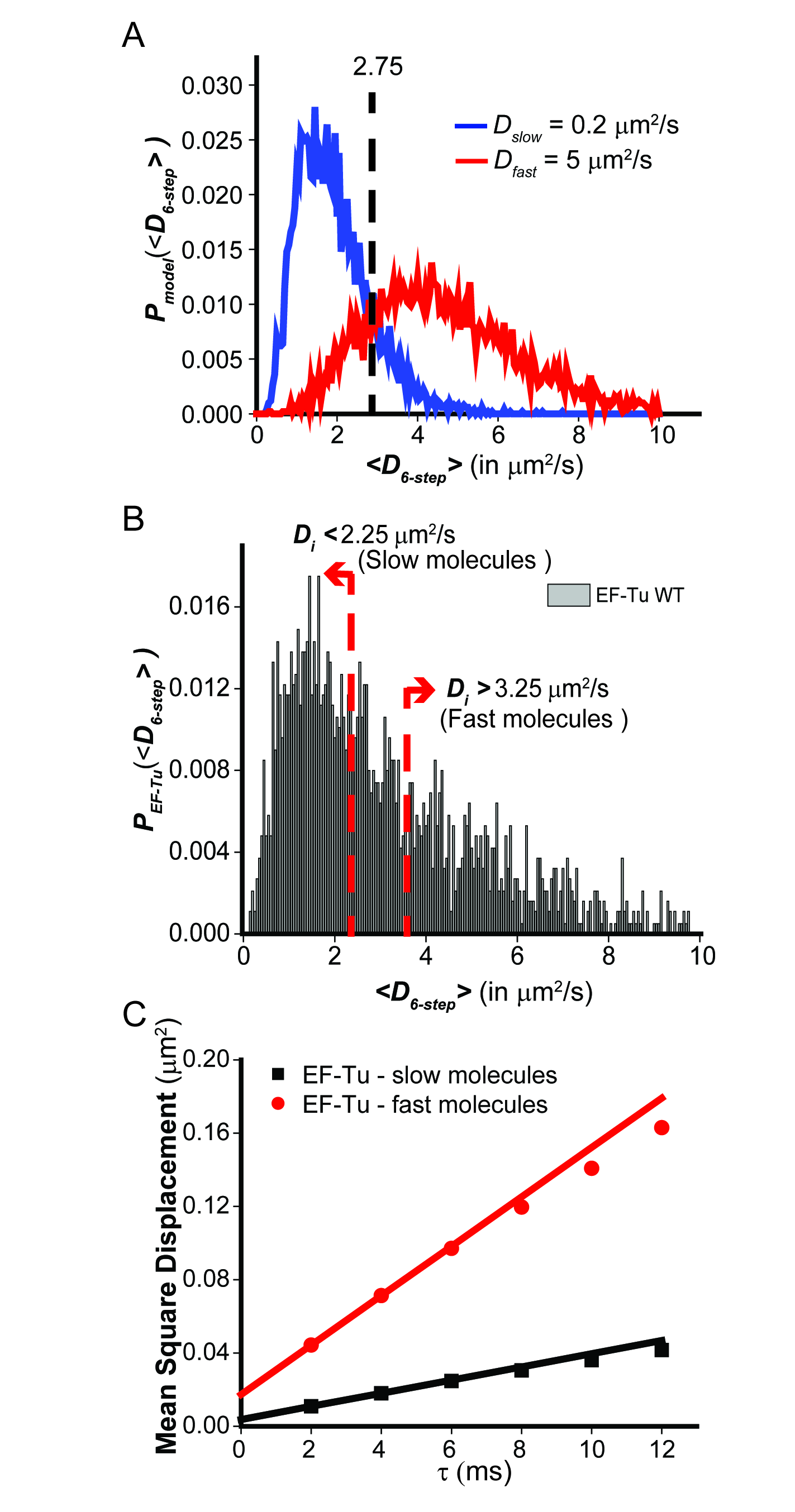

Supplement: FIG S6 [file mbo001183666sf6.tif]

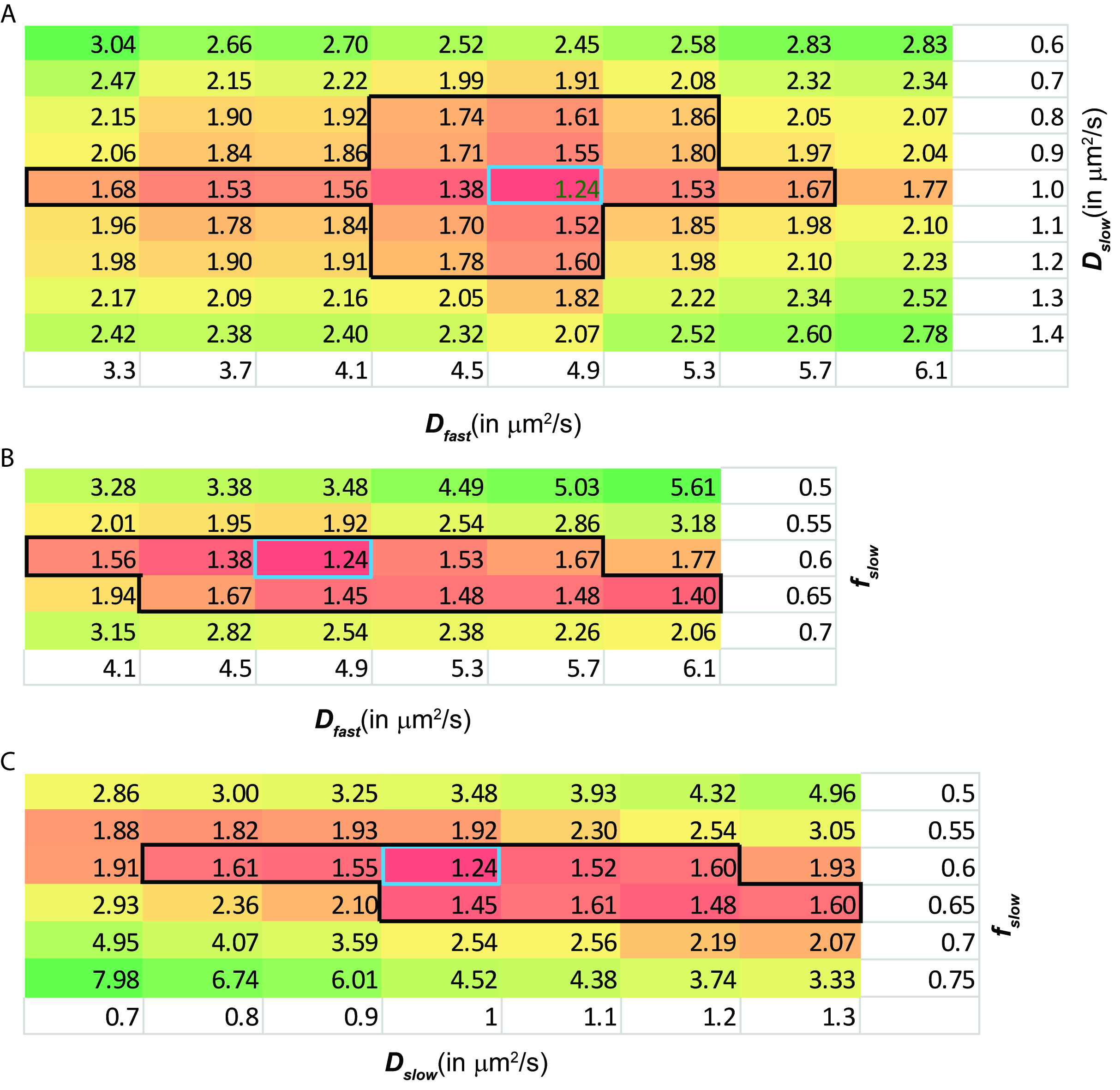

Supplement: FIG S7 [file mbo001183666sf7.tif]

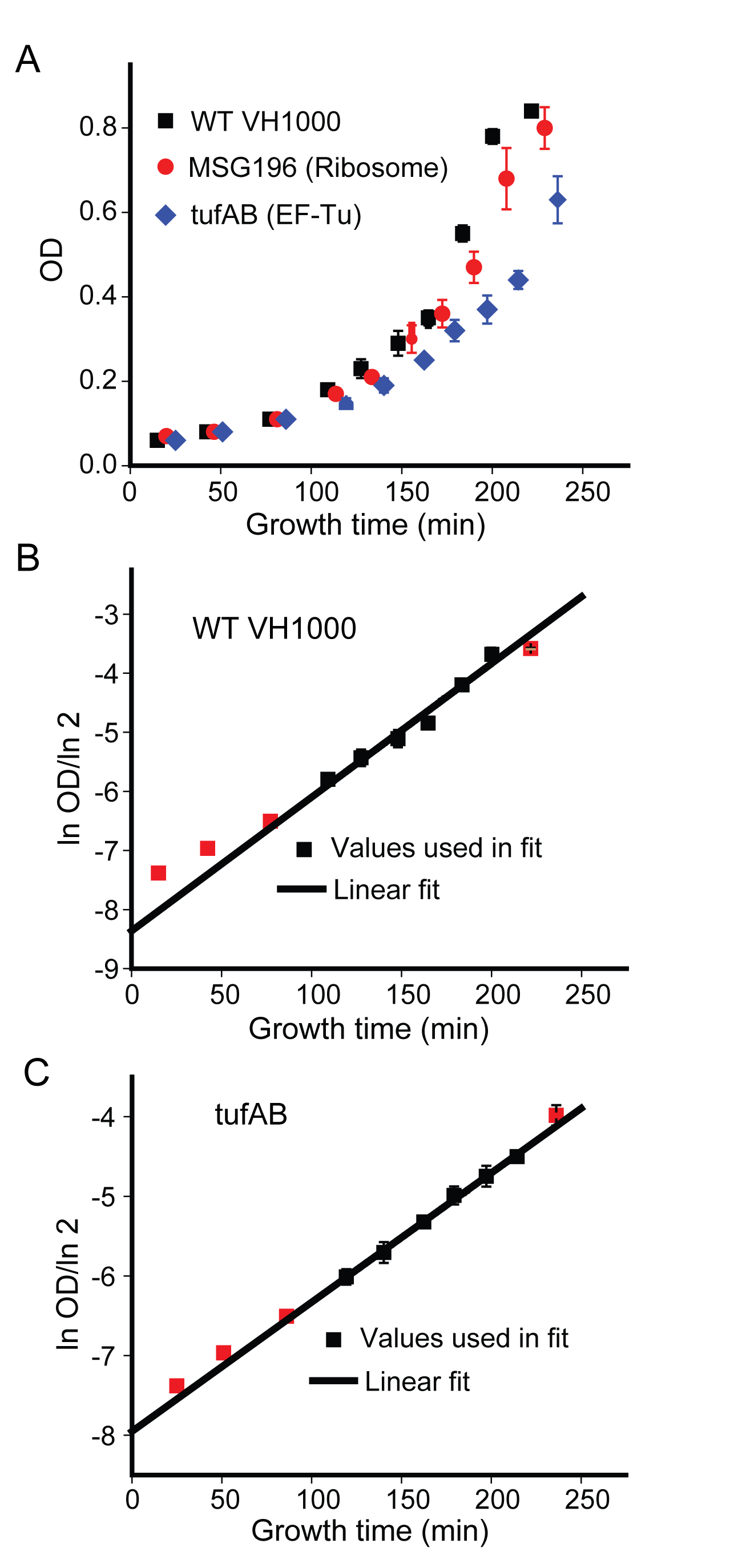

Supplement: FIG S8 [file mbo001183666sf8.tif]
