# Supplementary material for: Simultaneous Binding of Multiple EF-Tu Copies to Translating Ribosomes in Live Escherichia coli
Source: mBio. 2018 Jan 16;9(1):e02143-17. doi: 10.1128/mBio.02143-17 (PMC5770553; doi:10.1128/mBio.02143-17)
Supplement: TEXT S1 [file mbo001183666s1.pdf]

Supplemental Material

Bacterial strains and oligonucleotides used in this work

See Tables S1A and S1B below. Growth curves in Fig. S8.

Analysis of diffusive behavior

***Mean-square displacement plots MSD*(τ)**

The MSD as a function of lag time τ is given by $MSD(\tau) = <(\boldsymbol{r}(t+\tau) - \boldsymbol{r}(t))^2>$, where $\boldsymbol{r}(t)$ is the two-dimensional location of the particle at time t, τ is the lag time, and the average is taken over all times t and over many trajectories. MSD(τ) plots for the different species and imaging conditions are shown in Fig. 2. The slope of the first two points of an MSD(τ) plot provides a first estimate of the mean diffusion coefficient: D = slope/4. The MSD slope takes account of the localization error σ (1), but does not account for confinement effects. Even for 6 x 2 ms = 12 ms long trajectories, for rapidly diffusing species with $D \sim 5\ \mu m^2\text{-}s^{-1}$ confinement restricts diffusive trajectories and causes curvature of the MSD plot. This makes the diffusion coefficient from the two-point slope a lower bound on the true mean D. The trajectory analysis presented below is more accurate.

Estimation of dynamic localization error σ_{fast} and σ_{slow} for fast and slow EF-Tu molecules

Localization error in the single-step $P(r)$ distributions arises from the point-spread function (PSF) of the microscope, the finite number of photons detected per camera frame, and blurring of the images due to diffusive motion during each 2 ms camera frame (1, 2). While ribosomes and slow EF-Tu copies experience little diffusive blurring, the fast EF-Tu copies are blurred substantially. We reasoned that model Monte Carlo diffusive trajectories should involve larger dynamic localization error for fast EF-Tu copies than for slow EF-Tu copies.

This problem has been addressed in detail by Michalet (1). Suppose the best fit to the first two experimental points of a two dimensional mean-square displacement plot is given by the equation $MSD(\tau) = a + b\tau$, with b the slope and a the extrapolated intercept at lag time $\tau = 0$. Then the best estimate of the diffusion coefficient is $D = b/4$ and the best estimate of the dynamic localization error is $\sigma = ½ (a + 4Dt_E/3)^{1/2}$, where t_E is the exposure time per camera frame. We estimate that the diffusion coefficient of the fast (non-ribosome-bound) EF-Tu copies should be about 5 μm^2-s^{-1} (3, 4) and that of the ribosome-bound copies should be about 0.2 μm^2-s^{-1} (5). If so, the root-mean-square displacement in two dimensions of a fast EF-Tu copy during the 2-ms camera frame is ~200 nm, substantially larger than typical static localization errors in live *E. coli* single-molecule studies of slowly moving species labeled with fluorescent proteins. The rms displacement of a ribosome-bound EF-Tu copy is only ~60 nm, comparable to typical static localization errors.

For WT EF-Tu, the intercept of the MSD plot yields a mean localization error $\sigma = 60$ nm. To estimate the dynamic localization errors σ_{fast} and σ_{slow} for EF-Tu, we simulated six-step (6 x 2 ms) Monte Carlo diffusive trajectories in a model spherocylinder. One batch of 5000 trajectories had $D_{slow} = 0.2$ $\mu m^2/s$ and the other 5000 trajectories had $D_{fast} = 5$ $\mu m^2/s$; both used the mean localization error of 60 nm. These simulated trajectories were used to form the distribution of the mean of six one-step estimates of D:

$< D >_{6-step} = \frac{1}{24\tau}\sum_{i=1}^{6}\sqrt{(x_{i+1}-x_i)^2+(y_{i+1}-y_i)^2}$, where (x_{i+1}, y_{i+1}) and (x_i, y_i) are the coordinates of final and initial positions of each step. This distribution is shown in Fig. S6A. For WT EF-Tu, the analogous experimental distribution of $<D>_{6\text{-step}}$ is shown in Fig. S6B. The simulated distributions guided the choice of cutoffs that approximately distinguish slow from fast copies: <2.25 $\mu m^2/s$ for slow copies and >3.25 $\mu m^2/s$ for fast copies. Separate MSD plots for

these slow and fast experimental trajectories are shown in Fig. S6C. The intercepts of the linear fits to the first two data points gives the estimates σ_{slow} ~ 40 nm and σ_{fast} ~ 80 nm. These values were used to simulate trajectories for fitting the $P_{EF\text{-}Tu}(r)$ distributions to two static populations.

Monte Carlo simulations of diffusive trajectories

Many previous studies of single-molecule diffusion fit the experimental $P(r)$ distribution to a sum of analytical functions, with each component describing diffusion of the species in free space (6, 7). However, molecules diffusing rapidly in the *E. coli* cytoplasm suffer from confinement due to the spherocylindrical cell boundaries, a problem for which there is no analytical solution. Therefore, we simulated the behavior of each diffusive component from a large number of random walk trajectories that incorporate the dynamic localization error σ_{slow} or σ_{fast} and confinement effects within a model spherocylinder that mimics the dimensions of a typical *E. coli* cell in our growth conditions (tip-to-tip cell length = 4.3 μm and cell diameter = 0.9 μm). Each set of simulations models one diffusive state, with values of D and σ fixed. At t = 0, 5000 particles were randomly distributed within the cell volume. Each particle undergoes a random walk independent of other particle positions. To model each 2-ms camera image, three-dimensional microtrajectories (1000 steps of 2 μs each) were generated. At each time step, each particle chooses a displacement in each of three Cartesian directions. These displacements are chosen from a Gaussian distribution whose standard deviation corresponds to the state's three-dimensional diffusion coefficient D. In the rare event that a particle attempts to step outside of the cell boundaries, the displacement for that microstep is taken to be zero. The location of each particle during each camera frame is obtained as the centroid of the model microtrajectories in order to mimic the analysis procedure used for the experimental images. The appropriate dynamic localization error σ was then applied to each centroid location in both x and y coordinates by sampling a Gaussian distribution with standard deviation σ. By adding the error

to the centroid position we obtain the model “measured” location for each 2 ms camera frame. The *x* and *y* coordinates of each measured location are stored for further analysis. For the next model camera frame, each particle continues to make microsteps in 3D starting from the endpoint of the previous camera frame. Model trajectories for EF-Tu use the appropriate estimated value of σ_{slow} or σ_{fast}, determined as described above.

By connecting the sequence of simulation locations over seven frames, we form 5000 model trajectories for each relevant value of *D*. These trajectories are used to compute model-based, numerical one-step probability distributions $P_{model}(r;D)$ that are the model functions for the least-squares analysis of the corresponding experimental distributions.

Fitting of single-step P(r) distributions to static, two-state models

For each species in each experimental condition, a large set of trajectories that lasted for 6 steps or longer were selected for analysis. Longer trajectories were truncated to 6 steps. The 6-step trajectories were then sliced into individual steps. The single-step displacements $r_i = \sqrt{(x_{i+1} - x_i)^2 + (y_{i+1} - y_i)^2}$ were pooled to form the distribution $P(r)$, as in Figs. 3A and 4C. We typically attempt to fit the experimental distribution $P(r)$ in a least-squares sense to a single population or to a weighted average of two static populations. For a one-state model the only fitting parameter is *D*. For unconstrained models including two static (non-exchanging) states, the fitting function is the linear combination $P_{model}(r) = f_{slow}P(r;D_{slow}) + (1 - f_{slow})P(r;D_{fast})$. Here the three fitting parameters are D_{fast}, D_{slow}, and the fractional population f_{slow}, which in turn fixes $f_{fast} = (1 - f_{slow})$. For all our fitting procedures, D_{fast} ranged from 0.1 to 12 µm^2/s with interval of 0.1 µm^2/s and D_{slow} ranged from 0.05 to 3 µm^2/s with interval of 0.05 µm^2/s.

We judge the goodness of fit by evaluating the reduced chi-square statistic:

$\chi_\nu^2 = \frac{1}{(N-\alpha)}\sum_{j=1}^{N}\frac{(h_j - y_j)^2}{s_j^2}$. Here *j* labels the *N* bins in the (unnormalized) $P(r)$ and $P_{model}(r)$

histograms, h_j is the number of experimental counts in bin j, y_j is the number of counts in bin j of the simulated $P_{model}(D_i)$ histogram, s_j^2 is the variance of the value in bin j, and α is the number of fitted parameters. We take $s_j^2 = h_j$ as the estimate of the variance, assuming Poisson statistics. A good fit to an adequately flexible *analytical* model function should have $\chi_v^2 \sim 1$. In our numerical procedure, both the experimental distribution $P(r)$ and the fitting function $P_{model}(r)$ have noise, so we would expect good fits to have χ_v^2 values somewhat larger than 1. The fitting results for each species in various conditions are summarized in the main text and in Table 1.

To estimate the uncertainty in the model parameters for two-state fits, we examined the 3D grid of χ_v^2 values generated from the unconstrained fits that varied all three parameters D_{fast}, f_{slow}, and D_{slow}. For WT EF-Tu, three two-dimensional planes passing through the values D_{fast} = 4.9 μm^2-s^{-1}, f_{slow} = 0.60, and D_{slow} = 1.0 μm^2-s^{-1} are shown in Fig. S7. We judged the fits to be qualitatively poor whenever the value of χ_v^2 exceeded the minimum value by 0.5 units or more (Fig. S7). The parameter error estimates in Table 1 cover this range of χ_v^2 values in each case.

Fitting of single-step $P_{EF\text{-}Tu}(r)$ distributions to static, three-state models

We briefly explored three-state models (slow, fast, and medium) of the WT EF-Tu $P(r)$ distribution of Fig. 3A. To make the search tractable, the slow and fast diffusion coefficients were fixed to 0.1 and 4.9 μm^2/s, mimicking ribosome-bound and free EF-Tu states. The slow and the medium fractions were varied in intervals of 0.05 and the medium fraction diffusion coefficient D_{medium} from 0.1-5 μm^2/s in intervals of 0.1 μm^2/s. The fast fraction was determined as $f_{fast} = 1 - f_{slow} - f_{medium}$. The best fit parameters were $f_{slow} = 0.20 \pm 0.05$, $f_{medium} = 0.45 \pm 0.05$, and D_{medium} = 1.8 ± 0.5 μm^2/s, with $f_{fast} = 0.35 \pm 0.05$ and $\chi_v^2 = 1.25$ (Fig. S1C). This is no better fit than the best two-state model, which returned $\chi_v^2 = 1.24$. For the three-state fit, $f_{slow} + f_{medium}$ =

0.65 is in sensible agreement with the two-state result f_{slow} = 0.60. These fits are not very different in content. The sum of the slow and medium components in the best three-state fit is quite similar to the slow component of the best two-state fit.

Statistics of aa-tRNA sampling

The time-averaged stoichiometry of EF-Tu and tRNA binding to a translating 70S ribosome can be estimated from the fraction of EF-Tu copies bound to ribosomes combined with copy number estimates from other studies. There are 61 different codons and 43 different aa-tRNA types (43 different ternary complexes) used by *E. coli* (8). Forty-eight codons match only one type of ternary complex, 12 match two types, and one matches three types. This means that the ribosome is usually testing and rejecting non-cognate or near-cognate aa-tRNAs. In turn, this means the A site is most frequently occupied by an aa-tRNA within its ternary complex, still tethered to L7/L12 (prior to GTP hydrolysis and ejection of EF-Tu).

Fully 40 unique codons are used with at least 1% frequency (9). For a given mRNA codon poised at the 30S decoding site, the average chance that a particular ternary complex carries a cognate (completely matching) aa-tRNA anticodon is roughly 1 in 40. How many different ternary complexes must be sampled before a cognate aa-tRNA is found? For simplicity, we assume that there are N different aa-tRNA types (N different anti-codons) all required with equal probability in the transcriptome and all equally represented in the pool of aa-tRNA. This enables calculation of a closed form for the mean number of trials required to find an anti-codon that matches the current mRNA codon at the ribosome A site. Let $p = 1/N$ be the probability of a match on a given trial. The index k denotes the number of individual trials required to achieve a codon match. If the sampling of the pool is random, then the probability $P(k)$ of finding the first match on the kth trial is the probability of failing to find a match for $(k - 1)$ successive trials,

which is $(1-p)^{k-1}$, times the probability of finding a match on the kth trial: $P(k)=(1-p)^{k-1}p$.

The average number of trials required to find a match is:

$$<k> = \sum_{k=1}^{\infty} kP(k) = \sum_{k=1}^{\infty} k(1-p)^{k-1}p$$

$$= p\sum_{k=1}^{\infty} k(1-p)^{k-1}.$$

Let $x = (1-p)$. Then:

$$<k> = (1-x)\sum_{k=1}^{\infty} kx^{k-1}$$

$$= (1-x)\frac{d}{dx}\sum_{k=1}^{\infty} x^k$$ This is the derivative of a geometric series, which can be summed:

$$= (1-x)\frac{d}{dx}\frac{x}{1-x} = \frac{1}{1-x} = \frac{1}{p}.$$

For $p = 1/N$, the mean number of trials required to find a match is N. If $N \sim 40$, on average roughly 40 ternary complexes must be sampled before finding a cognate codon.

Estimates of copy numbers and partitioning of EF-Tu and tRNA

Under the same growth conditions used here (30°C in EZRDM), we previously estimated ~50,000 30S ribosomal subunits per cell, some 80% of which (~40,000 copies) are engaged as translating, 70S ribosomes (10). Mean copy number estimates for EF-Tu, total tRNA, EF-G, and total aa-tRNA synthetase (Table S1C) were derived from the ribosome copy number and from literature values of the ratio of each species' copy number to that of ribosomes. It was not possible to match strains, growth conditions, growth rate, and temperature, so we chose to match the growth rate (~1 doubling/hr).

EF-Tu is the most abundant protein in *E. coli*, about 7-fold more abundant than ribosomes (11, 12) (~350,000 total EF-Tu copies). The two-state analysis of $P_{EF\text{-}Tu}(r)$ in Fig. 3A combined with the axial distribution of these slowly diffusing copies indicates that ~60% of EF-Tu copies (~210,000) locate in the 70S-rich regions of space, where they undergo rapid exchange between

the ribosome-bound state (80% of the time) and rapidly diffusing state (20% of the time) on a timescale of ~2 ms or less. At a given moment, this corresponds to ~170,000 ribosome-bound EF-Tu copies (0.8 x 0.6 x 350,000). The remaining ~180,000 EF-Tu copies comprise free ternary complexes and bare EF-Tu seeking an aa-tRNA partner. The ~170,000 ribosome-bound EF-Tu copies is four times the estimated number of translating, 70S ribosomes. In *E. coli*, there are four L7/L12 sites per 50S subunit (13, 14). Under the assumption that ternary complexes bind to the ribosome only when interacting with L7/L12 N-terminal domains (further justified below), we infer that the four L7/L12 sites are essentially saturated with EF-Tu copies. An equilibrium binding calculation (below) corroborates this conclusion.

This analysis ignores the fact that each time a cognate aa-tRNA binds to the A site and transfers its aminoacyl group to the growing peptide chain, elongation factor G (EF-G) must bind to the A site and hydrolyze GTP in order to drive translocation of the A site tRNA to the P site and the P site tRNA to the E site. EF-G uses the same ribosomal L7/L12 sites as EF-Tu for its initial binding step (15). A tethered EF-G presumably samples the A site in much the same way as a tethered ternary complex. The estimated copy number of EF-G is 6-7 times smaller than that EF-Tu (11), *i.e.*, the EF-G copy number is similar to that of the ribosomes. *In vitro* studies found that EF-Tu and EF-G have comparable binding constants to L7/L12 units (16). Accordingly, we suggest that the mean occupancy of the four L7/L12 sites is ~3.5 EF-Tu copies (7/8 x 4) and ~0.5 EF-G copies (1/8 x 4). This seems a reasonable fractional occupancy of EF-G to ensure that a cognate aa-tRNA at the A site need not wait long for the arrival of an EF-G(GTP) copy to drive translocation. There is only one type of EF-G vs 43 types of ternary complex, so each EF-G visit to an L7/L12 site will be much more effective than each ternary complex visit.

So far, we have estimated that averaged over time, each ribosome binds ~3.5 ternary complexes (3.5 EF-Tu and 3.5 tRNA) and 0.5 EF-G copies, all tethered to L7/L12 subunits. One

of the ternary complexes typically occupies the A site. Next we estimate the partitioning of total tRNA among different species. The total tRNA copy number in *E. coli* growing at 1–2 doublings/hr is ~7-fold larger than the ribosome copy number (17). This implies ~7 x 50,000 = 350,000 total tRNA per cell in our growth conditions, the same as the estimated EF-Tu copy number. We estimate that averaged over time, each translating ribosome binds one tRNA in the A site (usually tethered to an L7/L12 via an EF-Tu bridge), one in the P site, perhaps one-half copy in the E site (a compromise between “2-1-2” and “2-3-2” elongation models (18, 19)), and an additional 2.5 copies bound as ternary complexes to the other three L7/L12 sites. Thus the time-averaged number of tRNA copies bound to each translating ribosomes is about 5. The ~40,000 translating ribosomes per cell in our growth conditions would then bind ~200,000 tRNA copies at a given moment in time (~57% of all copies). On average, 3.5 of these 5 copies (140,000) are associated with EF-Tu as ternary complexes and the other 1.5 copies (60,000) are ribosome-bound at the P and E sites and not associated with EF-Tu. The remaining ~150,000 tRNA copies (~43%) are not ribosome-bound and should partition among three diffusive states: free tRNA shortly after leaving the ribosomal E site, tRNA being recharged by aa-tRNA synthetase, and tRNA within ternary complexes.

A simple equilibrium calculation indicates that a large majority of the 290,000 tRNA copies not engaged at the ribosomal P and E sites should form ternary complexes and that most of the 350,000 EF-Tu copies also occur as ternary complexes. The binding constant *in vitro* between tRNA and EF-Tu has been measured to be $K_b = 7.5\ \mu M^{-1}$ (20). Binding constants are expected to be larger in the crowded cytoplasm than in buffer solution (21). The volume of an average, ~4 μm long, 0.9 μm diameter spherocylindrical cell is $V_{cell} = 2.35\ \mu m^3$. For that volume, each 1000 copies corresponds to a concentration of 0.71 μM. As an illustrative calculation, suppose we mixed 350,000 free EF-Tu copies (248 μM) with 290,000 free tRNA copies (206 μM) in the

cytoplasm. The solution-phase binding constant then predicts that at equilibrium, there will be 289,000 ternary complexes (accounting for 82% of all EF-Tu), 60,000 free EF-Tu copies, and only 1,000 free tRNA copies. However, that calculation neglects the tRNA copies that are bound to the 20 types of aa-tRNA synthetase, whose aggregate copy number is ~50,000 (11). In the limit of saturation of the synthetases with tRNA, only 240,000 tRNA copies would be available for binding with EF-Tu to form ternary complexes. At equilibrium, a mixture of 350,000 EF-Tu with 240,000 tRNA would yield 240,000 ternary complexes and 110,000 free EF-Tu. Roughly 70% of the EF-Tu would still exist as ternary complexes. The tRNA copies would then occur as 240,000 ternary complexes, 60,000 P- and E-site copies, 50,000 complexes with the synthetase, and only 400 free tRNA copies. This is the estimate used in the main text. Free tRNA is a small minority population in either equilibrium estimate.

Assuming the smaller estimate of 240,000 ternary complexes, we can use a second binding equilibrium calculation to estimate the average number of ternary complexes bound to the four L7/L12 units on each translating ribosome. *In vitro*, the binding constant between a single L7/L12 unit and a ternary complex is $K_b = 0.1\ \mu M^{-1}$ (16). Again, this likely underestimates the binding constant *in vivo*. Assume the cytoplasm initially contained 240,000 ternary complexes (170 μM) and 40,000 translating ribosomes (28 μM). Further assume independent binding (no cooperativity) of four ternary complexes to the four L7/L12 units on each 70S ribosome (22). Then at equilibrium the average number of ternary complexes bound to each 70S ribosome is 3.8 out of a maximum of 4.

The binding model goes as follows. Let R stand for a 70S ribosome and X stand for a ternary complex. Equilibrium concentrations are denoted by square brackets; for simplicity, $x = [X]$.

There are four sequential binding equilibria, each with the same binding constant K and its own equilibrium equation:

$$R + X \leftrightarrow RX, \qquad [RX] = [R]\, K\, x \qquad (1)$$

$$RX + X \leftrightarrow RX_2, \qquad [RX_2] = [RX]\, K\, x = [R]\, K^2\, x^2 \qquad (2)$$

$$RX_2 + X \leftrightarrow RX_3, \qquad [RX_3] = [RX_2]\, K\, x = [R]\, K^3\, x^3 \qquad (3)$$

$$RX_3 + X \leftrightarrow RX_4, \qquad [RX_4] = [RX_3]\, K\, x = [R]\, K^4\, x^4. \qquad (4)$$

Let R_0 be the total ribosome concentration and X_0 be the total ternary complex concentration:

$$R_0 = [R] + [RX] + [RX_2] + [RX_3] + [RX_4] \qquad (5)$$

$$X_0 = x + [RX] + 2[RX_2] + 3[RX_3] + 4[RX_4] \qquad (6)$$

Substituting Eqs. 1-4 into equations 5 and 6 and solving for $[R]$ and for $(X_0 - x)$ yields two expressions for $[R]$:

$$[R] = R_0 / (1 + Kx + K^2x^2 + K^3x^3 + K^4x^4) \qquad \text{and} \qquad (7)$$

$$[R] = (X_0 - x) / (Kx + 2K^2x^2 + 3K^3x^3 + 4K^4x^4) \qquad (8)$$

For given values of K and the initial concentrations R_0 and X_0, equations 7 and 8 can be set equal to each other and solved for x, whose value then determines all the equilibrium concentrations. In practice, we solved the equation by trial and error using an Excel spreadsheet.

These rough estimates based on solution-phase binding constants may well underestimate the extent of complex formation, both for formation of ternary complexes from EF-Tu and aa-tRNA and for binding of ternary complexes to L7/L12 units. The estimates help to justify the assertion that at a given moment most EF-Tu copies exist as ternary complexes. They are also consistent with the assertion that the four L7/L12 sites on each 70S ribosome are essentially saturated with ~4 bound ternary complexes. Finally, they predict that only a very small fraction of tRNA, perhaps 1% or less, exists as the free, unbound species.

In vivo rate constants for binding and unbinding of ternary complexes

Finally, we can estimate two *in vivo* rate constants for direct comparison with the recent set of theoretical *in vivo* rate constants derived from a large set of *in vitro* measurements obtained under standardized experimental buffer conditions and temperature. Our interpretation of the value D_{slow} = 1.0 μm^2-s^{-1}, as a composite of 80% ribosome-bound copies and 20% free copies, suggests that the timescale for bound-free exchange *within the ribosome-rich regions* must be 2 ms or less. This constrains the ratio τ_{on}/τ_{off} = 4 and the sum $(\tau_{on} + \tau_{off}) \leq 2$ ms. For concreteness, assume that $(\tau_{on} + \tau_{off})$ = 2 ms, which yields the values τ_{off} = 0.4 ms and τ_{on} = 1.6 ms. The time τ_{off} is the mean search time for a ternary complex to find an empty L7/L12 site within the ribosome-rich regions.

First we use the pseudo-first-order rate τ_{off}^{-1} and the 70S ribosome concentration to estimate the effective bimolecular association rate constant k_1 for binding of a typical non-cognate ternary complex to an L7/L12 subunit of a 70S ribosome within the ribosome-rich regions. Suppose the 40,000 70S ribosomes occupy roughly half of the 2.4 μm^3 volume of the average cell; then the concentration of translating ribosomes within the ribosome-rich regions is [70S] ~ 55 μM. The effective bimolecular rate constant becomes $k_1 = \tau_{off}^{-1}/[70S] = 4.5 \times 10^7$ $M^{-1}s^{-1}$. This is remarkably fast, and if $(\tau_{on} + \tau_{off})$ were <2 ms, the estimate would be even larger. For comparison, the diffusion-limited bimolecular rate constant for ribosomal spheres of radius R_{ribo} = 10 nm and diffusion coefficient D_{ribo} = 0.1 μm^2/s colliding with ternary complex spheres with R_{TC} = 2 nm and D_{TC} = 5 μm^2/s is $k_{diff} = 4\pi N_0(R_{ribo} + R_{TC})(D_{ribo} + D_{TC}) = 3.2 \times 10^8$ $M^{-1}s^{-1}$, only sixfold larger than our estimated k_1. Roughly one in six close encounters between a ternary complex and a 70S ribosome, leads to a binding event. As suggested earlier (13), k_1 may be very fast due to the presence of four L7/L12 binding sites per ribosome and the flexibility of the

linkages between ribosome and the C-terminal domain of L7/L12. We call k_1 an effective rate constant because in many close encounters the ternary complex will find most L7/L12 binding sites already occupied by a ternary complex or an EF-G, unlike pseudo-first-order conditions used in kinetics assays. The theoretical *in vivo* estimate for κ_{on}* (Table 2 of Ref. (8)) at 1.07 doublings/hr and 37°C is 9.4 x 10^7 $M^{-1}s^{-1}$, twofold larger than our lower limit on k_1.

We can also compute the unimolecular dissociation rate of EF-Tu (typically part of a ternary complex) from the ribosome, $k_{-1} = \tau_{on}^{-1} \geq 625$ s^{-1} at 30°C with the theoretical *in vivo* rate for 1.07 doublings/hr at 37°C. The latter is $\omega_{off}* = 1700$ s^{-1} (Table 2 of Ref. (8)). Correcting from our 30°C to 37°C increases k_{-1} by a factor of 2.1: k_{-1}(37°C) = 2.1 k_{-1}(30°C) ≥ 1250 s^{-1}. The effects of temperature on k_{-1} were derived from the T-dependence of the experimental *in vitro* rate constants in Table 1 of Ref. (8): k_{-1} = 85 s^{-1} at 20°C and 700 s^{-1} at 37°C. We assumed the Arrhenius temperature dependence with A-factor independent of T: $k_{-1}(T) = A \exp[-E_a/RT]$, with E_a the activation energy and R the gas constant.

Supplemental References

1. Michalet X. 2010. Mean Square Displacement Analysis of Single-Particle Trajectories with Localization Error: Brownian Motion in Isotropic Medium. Phys Rev E Stat Nonlin Soft Matter Phys 82:041914-041914.

2. Thompson RE, Larson DR, Webb WW. 2002. Precise nanometer localization analysis for individual fluorescent probes. Biophys J 82:2775-2783.

3. Nenninger A, Mastroianni G, Mullineaux CW. 2010. Size dependence of protein diffusion in the cytoplasm of *Escherichia coli*. J Bacteriol 192:4535-40.

4. Bakshi S, Bratton BP, Weisshaar JC. 2011. Subdiffraction-limit study of Kaede diffusion and spatial distribution in live *Escherichia coli*. Biophys J 101:2535-44.

5. Mohapatra S, Choi H, Ge X, Sanyal S, Weisshaar JC. 11 May 2017. Spatial Distribution and Ribosome-binding Dynamics of EF-P in Live *E. coli*. mBio doi:10.1128/mBio.00300-17.

6. Chen T-Y, Santiago AG, Jung W, Krzemiński Ł, Yang F, Martell DJ, Helmann JD, Chen P. 2015. Concentration- and chromosome-organization-dependent regulator unbinding from DNA for transcription regulation in living cells. Nat Commun 6:7445.

7. Plochowietz A, Farrell I, Smilansky Z, Cooperman BS, Kapanidis AN. 2017. In vivo single-RNA tracking shows that most tRNA diffuses freely in live bacteria. Nucleic Acids Res 45:926-937.

8. Rudorf S, Thommen M, Rodnina MV, Lipowsky R. 2014. Deducing the kinetics of protein synthesis in vivo from the transition rates measured in vitro. PLoS Comput Biol 10:e1003909.

9. Maloy S, V. Stewart, and R. Taylor. 1996. Genetic analysis of pathogenic bacteria. Cold Spring Harbor Laboratory Press, NY.

10. Bakshi S, Choi H, Weisshaar JC. 2015. The spatial biology of transcription and translation in rapidly growing *Escherichia coli*. Front Microbiol 6:636.

11. Schmidt A, Kochanowski K, Vedelaar S, Ahrne E, Volkmer B, Callipo L, Knoops K, Bauer M, Aebersold R, Heinemann M. 2016. The quantitative and condition-dependent *Escherichia coli* proteome. Nat Biotechnol 34:104-10.

12. Klumpp S, Scott M, Pedersen S, Hwa T. 2013. Molecular crowding limits translation and cell growth. Proc Natl Acad Sci U S A 110:16754-9.

13. Diaconu M, Kothe U, Schlunzen F, Fischer N, Harms JM, Tonevitsky AG, Stark H, Rodnina MV, Wahl MC. 2005. Structural basis for the function of the ribosomal L7/12 stalk in factor binding and GTPase activation. Cell 121:991-1004.

14. Voorhees RM, Ramakrishnan V. 2013. Structural basis of the translational elongation cycle. Annu Rev Biochem 82:203-36.

15. Savelsbergh A, Mohr D, Kothe U, Wintermeyer W, Rodnina MV. 2005. Control of phosphate release from elongation factor G by ribosomal protein L7/12. EMBO J 24:4316-4323.

16. Savelsbergh A, Mohr D, Wilden B, Wintermeyer W, Rodnina MV. 2000. Stimulation of the GTPase Activity of Translation Elongation Factor G by Ribosomal Protein L7/12. J Biol Chem 275:890-894.

17. Dong H, Nilsson L, Kurland CG. 1996. Co-variation of tRNA Abundance and Codon Usage in *Escherichia coli* at Different Growth Rates. J Mol Biol 260:649-663.

18. Uemura S, Aitken CE, Korlach J, Flusberg BA, Turner SW, Puglisi JD. 2010. Real-time tRNA transit on single translating ribosomes at codon resolution. Nature 464:1012-7.

19. Chen C, Stevens B, Kaur J, Smilansky Z, Cooperman BS, Goldman YE. 2011. Allosteric vs. spontaneous exit-site (E-site) tRNA dissociation early in protein synthesis. Proc Natl Acad Sci U S A 108:16980-5.

20. Abdulkarim F, Ehrenberg M, Hughes D. 1996. Mutants of EF-Tu defective in binding aminoacyl-tRNA. FEBS Letters 382:297-303.

21. Phillip Y, Schreiber G. 2013. Formation of protein complexes in crowded environments – From in vitro to in vivo. FEBS Letters 587:1046-1052.

22. Dill K, Bromberg S. 2010. Molecular Driving Forces: Statistical Thermodynamics in Biology, Chemistry, Physics, and Nanoscience. Garland Science.

23. Reeh S, Pedersen S. 1978. Regulation of *Escherichia coli* elongation factor synthesis *in vivo*. Gene Expression (11th FEBS Meeting Copenhagen 1977), eds Clark BFC, Klenow H, Zeuthen J (Pergamon, Oxford),:89–98.
